# Genome Majority Vote Improves Gene Predictions

**DOI:** 10.1371/journal.pcbi.1002284

**Published:** 2011-11-17

**Authors:** Michael E. Wall, Sindhu Raghavan, Judith D. Cohn, John Dunbar

**Affiliations:** 1 Computer, Computational, and Statistical Sciences Division, Los Alamos National Laboratory, Los Alamos, New Mexico, United States of America; 2 Center for Nonlinear Studies, Los Alamos National Laboratory, Los Alamos, New Mexico, United States of America; 3 Theoretical Division, Los Alamos National Laboratory, Los Alamos, New Mexico, United States of America; 4 Department of Computer Science, The University of Texas at Austin, Austin, Texas, United States of America; 5 Bioscience Division, Los Alamos National Laboratory, Los Alamos, New Mexico, United States of America; The Centre for Research and Technology, Hellas, Greece

## Abstract

Recent studies have noted extensive inconsistencies in gene start sites among orthologous genes in related microbial genomes. Here we provide the first documented evidence that imposing gene start consistency improves the accuracy of gene start-site prediction. We applied an algorithm using a genome majority vote (GMV) scheme to increase the consistency of gene starts among orthologs. We used a set of validated *Escherichia coli* genes as a standard to quantify accuracy. Results showed that the GMV algorithm can correct hundreds of gene prediction errors in sets of five or ten genomes while introducing few errors. Using a conservative calculation, we project that GMV would resolve many inconsistencies and errors in publicly available microbial gene maps. Our simple and logical solution provides a notable advance toward accurate gene maps.

## Author Summary

The genetic code tells us precisely how a DNA sequence will be translated into a protein. However, it is more difficult to identify where translation will start and stop in the entire length of an organism's genome sequence. Computer software can predict where the start sites are, and this is successful most of the time; however, errors do occur. We hypothesized that some errors might be corrected by comparing predictions for the genome sequences of closely related organisms. This correction scheme seems especially appropriate for bacterial genomes: not only is protein production in bacteria simpler than in higher organisms, but hundreds of bacterial DNA sequences are now available, and many of these are closely related. To test the hypothesis, we developed a method to detect whether a gene's start site is inconsistent with the majority of equivalent genes in a set of related bacterial genomes. The method then modifies the start if it can be made consistent with the majority of genomes. Our tests show this majority vote method improves the accuracy of gene start sites. Application of the method to existing bacterial genomes should eliminate many inconsistencies and correct a large number of errors.

## Introduction

All of genomics depends on accurate identification of coding regions. Most gene boundaries are predicted using computational methods, and only a tiny fraction have been verified experimentally. Unfortunately, the accuracy of current gene-finding algorithms is not perfect. Error rates for the most common algorithms—Glimmer3 [1], GeneMark [2], and Prodigal [3]—currently range from 1.5%–17.6% [3]. Gene prediction errors alter protein sequences and intergenic regions (IGRs). Changes in protein sequence influence calculations of similarities, phylogenetic analyses, and can lead to errors in function annotation. Changes in IGRs affect a suite of other predictions, such as operon structure, regulatory motifs, and comparison of regulatory regions among genomes. Changes in gene boundaries also affect microarray design and interpretation of microarray data [4].

Gene-prediction error is a well-recognized problem [5] but the full extent of gene prediction errors from current computational methods is unknown. Recent studies yielded insight into the problem for bacterial genomes. Pallejà, Harrington, & Bork [6] found nearly a thousand examples of spurious gene overlaps (a gene stop being downstream of the following gene's start) in 338 bacterial genomes. Recently, we noted inconsistencies in gene start sites among 53% of the orthologous gene sets across the *Burkholderia* genus [7]. Although we expected real biological variation to yield some inconsistencies in gene starts, many inconsistencies for the *Burkholderia* genus included predictions of alternative starts in regions of nearly identical sequence and likely represented errors. We found most of these start site inconsistencies could be resolved by choosing alternative start sites for one or more of the orthologous genes, improving comparisons of IGRs across the genus. We and others have speculated (either implicitly or explicitly) that efforts to improve consistency of gene boundaries among orthologs can also improve the accuracy of gene predictions [7], [8], [9]. However, this hypothesis has not yet been tested.

Here, we test this idea using a set of validated *Escherichia coli* genes. We provide, for the first time, quantitative evidence showing that consistency increases accuracy. We discuss the significance of our results in the context of gene prediction methods that make use of multiple genomes, and find that our method is distinguished both by its effective use of larger numbers of genomes, by its simplicity and modularity, and by its use of contemporary (not older and error-ridden) gene-predictions. To our knowledge, the method is the only tool available for non-specialists to solve the routine problem of refining the accuracy of extant gene maps in public databases.

## Results/Discussion

### Motivation for improving gene predictions using a majority vote

Gene finding programs need to evaluate several possible start sites for each gene. The programs occasionally make mistakes and pick the wrong start site. If mistakes for orthologous genes in different genomes are uncorrelated (an unrealistic assumption, but useful as a reference point for algorithm development), then if less than half of the predicted starts are wrong, they might be corrected by a majority vote.

To formulate the majority vote idea mathematically, consider a set of *N* orthologous genes with experimentally verified start sites that have consistent positions in a multiple sequence alignment. If the probability of a gene finder predicting the wrong start site for any of the orthologs is *e*, and if the predictions for different genomes are independent, then the probability *p_i_* of finding *i* errors among all of the orthologs is given by a binomial distribution,
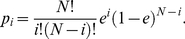
(1)


The probability of finding at least one error among all of the orthologs is
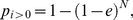
(2)and the probability of the majority of the orthologs containing an error is
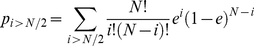
(3)


For example, let the probability of predicting an incorrect start site be *e* = 0.05, at the low end of the range of error rates for common gene finders [3]. If the number of orthologous genes *(N)* is 5, the chance of at least one error, 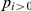, in the ortholog set is 22.6%, but the chance that the majority of orthologs are erroneous (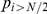) is only 0.12%. The mean error rate 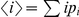 is 0.25 across all number of errors for five orthologs, and is 0.0035 across *i*>2. In this scenario, choosing a globally consistent site where a majority of the original predictions coincide is expected to correct 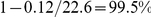 of the inconsistent ortholog sets, and to correct 1−0.0035/0.25 = 98.6% of the individual genes that have prediction errors.

The above model illustrates that typical gene prediction error rates can lead to double-digit inconsistencies in ortholog sets (e.g. in the above case, a 5% error rate led to a 22.6% inconsistency rate). It also illustrates the ability of a majority vote to decrease errors and thereby increase accuracy through increasing consistency. The increase in accuracy requires that the error rate for a single gene start be less than 50%. This prerequisite is satisfied by modern gene calling software, for which reported error rates range from 1.5% to 17.6% [3].

### Genome Majority Vote algorithm

Although the above model gives clear and quantitative insight into how comparative genomics might improve the accuracy of gene maps, it is merely a reference point and does not consider the important effects of real biological variation and correlated errors. In our previous study of gene start site consistency in the *Burkholderia* genus [7], we noted that ortholog sets in which a majority of the start sites did not coincide were likely to represent biological variation, whereas ortholog sets in which a majority of the start sites coincided were likely to represent errors. To determine whether a majority vote scheme might decrease start site errors in real gene maps, we developed a Genome Majority Vote (GMV) algorithm and applied it to a conservative test case: gene maps from *E.coli* and close relatives.

The GMV algorithm works as follows. For a given set of orthologous genes, if the positions of the start sites already coincide in a multiple sequence alignment, they are accepted. If they do not coincide, a start position is sought which is consistent for the majority of the genes and for which there is a reasonable alternative start site for the remaining genes in the set. If such a position is found, it is accepted, and the predictions are changed for the outlying genes. Otherwise, no start site prediction is made for the ortholog set.

**Figure 1:**
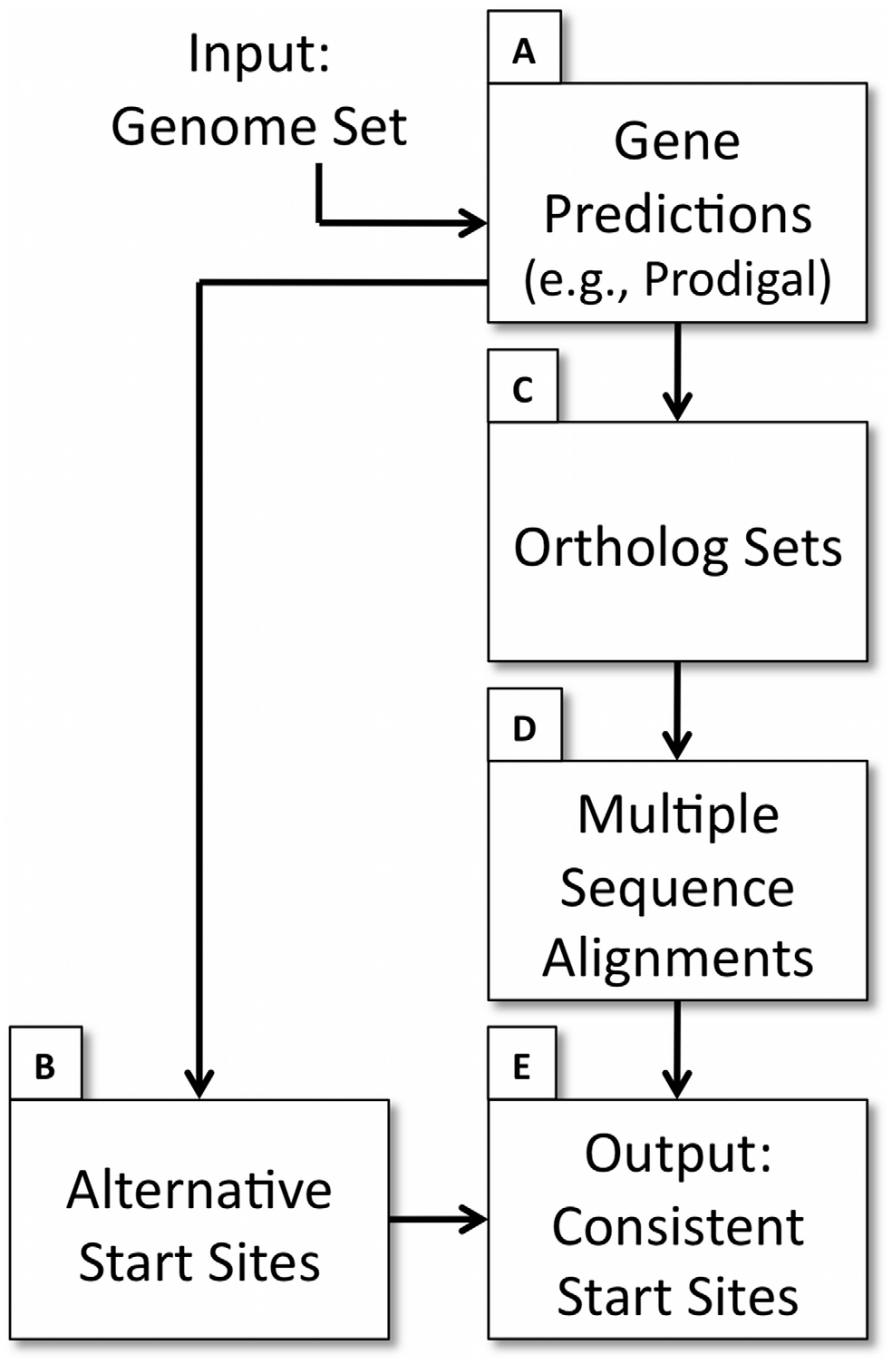
Flow diagram for the pipeline implementing the Genome Majority Vote algorithm. Individual steps A–E are explained in the text (Methods).

**Figure 2:**
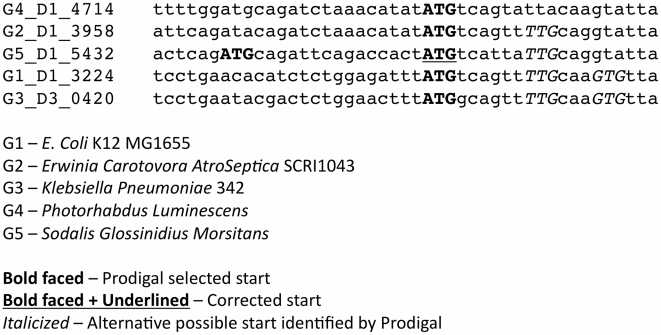
Example of a GMV modification of gene starts that is typical in terms of ortholog sequence identity, change in the length of the gene, and the start codon before and after the change.

We implemented GMV in the pipeline illustrated in Fig. 1. The input of the pipeline is a set of genome FASTA files. The output is a set of gene predictions for each genome after enforcing consistency using a genome majority vote (GMV) algorithm. A typical GMV correction is illustrated in Fig. 2. Details of the pipeline are described in the Methods section.

### Conservative approach to evaluation of the GMV algorithm

Any gene-calling software can in principle be used as a front end to provide input gene calls to GMV. Here we used Prodigal [3] predicted gene maps for *E. coli* and close relatives as a starting point for GMV evaluation. This choice solved two problems. First, Prodigal conveniently provided a list of reasonable alternative start sites for each gene, simplifying the comparison and reassignment of possible start sites among genomes. Second, Prodigal provided gene maps with fewer prediction errors compared to existing GenBank annotations that were obtained from older, more error prone versions of gene finders like Glimmer2. Prodigal is reportedly the most robust gene finder for diverse genomes [3]. Therefore, our use of Prodigal gene maps instead of Glimmer3 [1], GeneMark [2], or older annotations appeared to be the most logical and conservative approach.

Because Prodigal gene maps are likely to be more accurate than most of the gene maps currently in GenBank [3], using Prodigal gene maps to test the performance of GMV in correcting errors should provide conservative estimates of performance. We reported previously that Genbank maps for *Burkholderia* species were more inconsistent than Prodigal maps [7]; we show similar results in a later section of this paper for a set of *E. coli* genomes of comparable diversity. Use of more error-prone gene maps—either from other gene finders or from genomes that are more problematic for gene prediction—would be expected to inflate the number of observed inconsistencies among orthologs and the projected impact of applying the GMV algorithm.

To evaluate the performance of the algorithm, we created eight genome test sets that varied in size and diversity (Supplementary Table S1, Supplementary Figs. S_1_–S_8_). Each set contained either 5 or 10 genomes and included *E. coli* K-12 MG1655 as the reference genome. The sets represented low, medium, high, or very high diversity. We used a set of 871 experimentally validated *Escherichia coli* K12 MG1655 genes downloaded from the EcoGene web site [10] (http://ecogene.org) as a standard to determine error rates. A gene prediction was classified as erroneous if the translational start site differed from that of the validated gene; no errors were detected in translational stop sites.

### GMV increases gene start site consistency

Among the eight test sets, 5.9% to 61.8% of the ortholog sets had inconsistencies. The majority vote rule improved consistency for 13.2% to 51.9% of the ortholog sets (Table 1). The impact varied depending on the number and diversity of genomes in the test sets. The impact was highest for the medium diversity sets.

**Table 1:** Consistency statistics for ortholog sets.

	5 genomes^a^				10 genomes^a^			
	Low	Medium	High	Very High	Low	Medium	High	Very High
Total # of ortholog sets generated in the pipeline	3633	2446	1414	988	3271	2133	1317	380
# of ortholog sets for which Prodigal starts were initially inconsistent^b^	213 (5.9%)	536 (21.9%)	574 (40.6%)	547 (55.4%)	251 (7.7%)	614 (28.8%)	634 (48.1%)	235 (61.8%)
# of ortholog sets for which Prodigal starts were already consistent^b^	3420 (94.1%)	1910 (78.1%)	840 (59.4%)	441 (44.6%)	3020 (92.3%)	1519 (71.2%)	683 (51.9%)	145 (38.2%)
# of inconsistent ortholog sets that were made consistent by GMV^c^	74 (34.7%)	278 (51.9%)	204 (35.5%)	74 (16.8%)	89 (35.5%)	286 (46.6%)	227 (35.8%)	31 (13.2%)
# of ortholog sets with consistent starts after GMV^b^	3494 (96.2%)	2188 (89.5%)	1044 (73.8%)	515 (52.1%)	3109 (95.0%)	1805 (84.6%)	910 (69.1%)	176 (46.3%)
# of ortholog sets with at least one consistent start^b^	3626 (99.8%)	2428 (99.3%)	1326 (93.8%)	863 (87.3%)	3269 (99.9%)	2098 (98.4%)	1215 (92.3%)	310 (81.6%)

a The genomes in each set are listed in Supplementary Table S1.

b Percentage is with respect to total # of ortholog sets generated in the pipeline.

c Percentage is with respect to # of ortholog sets for which Prodigal starts were initially inconsistent.

The maximum level of consistency that could theoretically be imposed ranged from 81.6% to 99.9% (Table 1, last row). The difference between the theoretical maximum and the levels achieved by GMV ranged from 3.6% to 35.2%; these differences increased monotonically with the diversity of the genome test sets. The range of differences encompasses a value of 18% calculated from the results for five *Burkholderia* genomes [7], demonstrating the general consistency of the previous results with the results presented here. The ortholog sets not revised by GMV involve choosing alternative start sites for the majority of genes. These ortholog sets might represent real biological variation in the location of gene start sites, as we discussed previously [7]. In contrast, GenePRIMP [9] also uses homologs to identify potential start site prediction errors, but does not appear to have a mechanism that could distinguish errors from true biological variation.

### GMV changes typically preserve start codons

Among the ortholog sets revised by GMV, ATG was the most common start codon, as expected (Table 2). We calculated statistics for start codon changes for the medium and high diversity genome test sets, which accounted for the largest number of revised ortholog sets. The start codon identity was preserved in 69%–75% of GMV revisions. The start codon distribution for ortholog sets before revision by GMV, calculated as the mean among four test sets, was approximately 87% ATG, 9% GTG, and 4% TTG. After revision, the distribution was 79.5% ATG, 14.5% GTG, and 6% TTG.

**Table 2:** Codon change statistics for GMV start site changes in medium and high diversity genome test sets.

		5 genomes		10 genomes	
Codon before change	Codon after change	Medium	High	Medium	High
ATG	ATG	243	184	354	249
ATG	GTG	47	26	66	41
ATG	TTG	16	10	33	26
GTG	ATG	31	15	42	22
GTG	GTG	8	5	5	7
GTG	TTG	0	1	0	1
TTG	ATG	9	10	14	11
TTG	GTG	3	1	7	5
TTG	TTG	0	0	1	1
Total Changes		357	252	522	363
Same codon		251	189	360	257
Different codon		106	63	162	106

### GMV increases gene prediction accuracy

Before applying GMV, we first note evidence of an association between consistency and accuracy of gene start sites among orthologs. Among ortholog sets with consistent start sites, the *E. coli* start site accuracy ( = 100% – [error rate]) ranged from 96% to 100% (Table 3, row 5) in low to high diversity genome test sets. The start site accuracy was lower for orthologs with inconsistent start sites, ranging from 69.2% to 91.8% (Table 3, row 6). Overall, the error rate for consistent start sites was about 15% lower than the error rate for inconsistent start sites (Table 3, subtract row 6 from row 5 and calculate the mean). This observation supports the notion that the pursuit of consistency can improve accuracy.

**Table 3:** Validation statistics for ortholog sets.

	5 genomes				10 genomes			
	Low	Medium	High	Very High	Low	Medium	High	Very High
# of ortholog sets for which *E. coli* validation was available	833	683	457	274	800	618	414	129
# of ortholog sets for which *E. coli* validation was available and for which Prodigal predictions were already consistent^a^	825 (99.0%)	613 (89.8%)	382 (83.6%)	245 (89.4%)	787 (98.4%)	546 (88.3%)	329 (79.5%)	107 (82.9%)
# of ortholog sets for which *E. coli* validation was available and for which Prodigal predictions were inconsistent^a^	8 (0.96%)	70 (10.2%)	75 (16.4%)	29 (10.6%)	13 (1.63%)	72 (11.7%)	85 (20.5%)	22 (17.1%)
# of ortholog sets with start sites matching a validated *E. coli* start^a^	799 (95.9%)	664 (97.2%)	444 (97.2%)	271 (98.9%)	769 (96.1%)	602 (97.4%)	406 (98.1%)	126 (97.7%)
# of ortholog sets with start sites matching a validated *E. coli* start and for which Prodigal predictions were already consistent^b^	792 (96.0%)	609 (99.3%)	381 (99.7%)	245 (100%)	760 (96.6%)	544 (99.6%)	328 (99.7%)	107 (100%)
# of ortholog sets with start sites matching a validated *E. coli* start and for which Prodigal predictions were inconsistent^c^	7 (87.5%)	55 (78.6%)	63 (84.0%)	26 (89.7%)	9 (69.2%)	58 (80.6%)	78 (91.8%)	19 (86.3%)

a Percentage is with respect to total # of ortholog sets.

b Percentage is with respect to # of ortholog sets for which *E. coli* validation was available and for which all Prodigal predictions were already consistent. This represents accuracy of the consistent subset.

c Percentage is with respect to # of ortholog sets for which *E. coli* validation was available and for which Prodigal predictions were inconsistent. This represents accuracy of the inconsistent subset.

**Figure 3:**
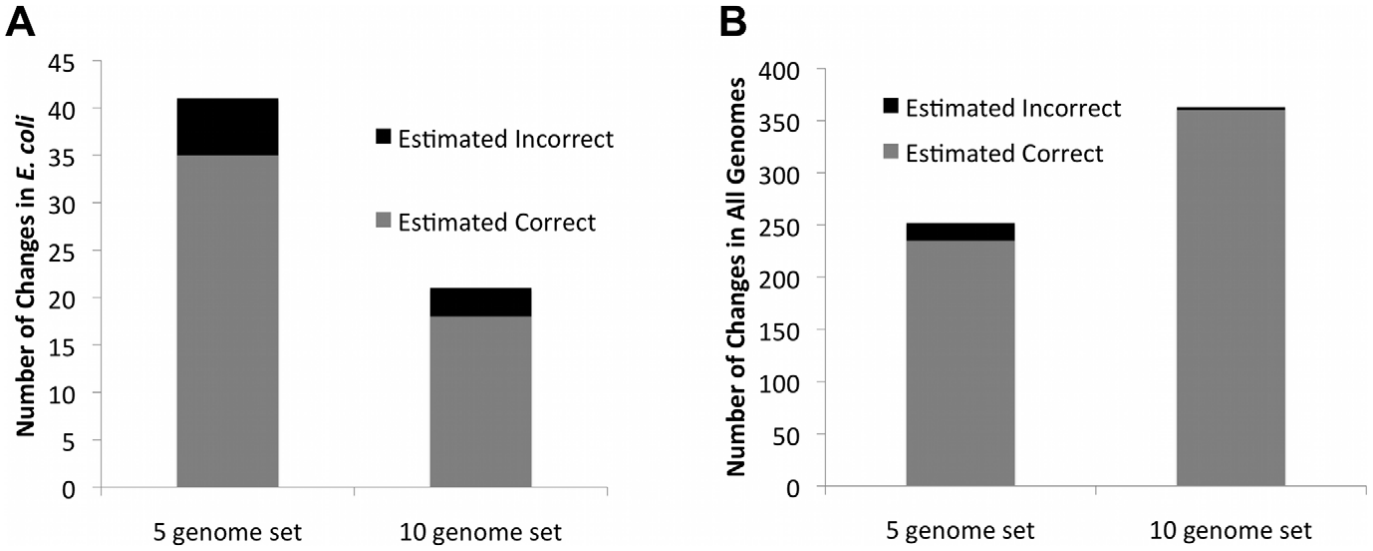
Impact of gene prediction changes in high diversity genome sets. Number of correct and incorrect changes are estimated using validated starts in *E. coli*, as described in the text. A) *E. coli*; B) All genomes.

The GMV pipeline corrected the most errors when applied to the high and medium diversity test sets (Table 4). Error rates (*i.e.* rates of inappropriate corrections) were lower for the high diversity sets, and more corrections were produced for the medium diversity sets. In the high diversity 5-genome test set, GMV yielded 41 modifications in *E. coli*, which included 13 genes with validated start sites. For the 13 ground truth positives (GP), GMV corrected 11 errors but also incorrectly shifted 2 previously correct start sites. In other words, GMV yielded 11 true positives (*TP*) and 2 false positives (*FP*) for this data set. The sensitivity was *S* = *TP*/*GP* = 0.846, and the error rate was *E* = *FP*/(*TP*+*FP*) = 0.154. Applying this error rate to all 41 modified starts in *E. coli* yields an estimated 35 correct changes and 6 incorrect changes (Fig. 3). For the other genomes in the test set, GMV changed a total of 252 start sites, 88 of which were in ortholog sets for which *E. coli* validation information was available. The positions of 82 of the 88 changes coincided with a validated *E. coli* start site, while the other 6 were erroneous. These numbers yield an error rate of 6/82 = 0.07. This is about half the error rate calculated for *E. coli* alone and may be more representative because it was derived from a larger sample size (n = 82, compared to n = 13); if the *E. coli* predictions had yielded 1 false positive instead of 2, the *E. coli* rate would also have been 0.07, illustrating the sensitivity of statistics to small changes when the sample size is small. Applying the 0.07 error rate to all 252 changes that GMV made, we expect 235 of these to be correct and 17 to be incorrect changes. With the 10-genome high-diversity test set, there were more modifications, and the error rate was lower: GMV changed a total of 363 start sites, and only 3 of the changes were predicted to be incorrect. For the medium-diversity test sets, there were more modifications at the cost of a higher error rate: with 5 genomes, GMV changed a total of 357 start sites, 30 of which are predicted to be incorrect; with 10 genomes, 522 start sites were changed, 54 of which are predicted to be incorrect.

**Table 4:** Validation statistics for GMV algorithm corrections to Prodigal gene maps.

	5 genomes				10 genomes			
	Low	Medium	High	Very High	Low	Medium	High	Very High
# of ortholog sets with an incorrect *E. coli* start (*GP*)	34	19	13	3	31	16	8	3
# of corrected validated starts in *E. coli* (*TP*)	0	9	11	3	1	8	7	3
# of *E. coli* errors introduced (*FP*)	1	2	2	1	0	0	0	0
Error Rate (*E* a)	1.00	0.182	0.154	0.25	0.5^b^	0.111^b^	0.125^b^	0.25^b^
Sensitivity (*S* c)	0	0.474	0.846	1.0	0.032	0.5	0.875	1.00
Total # of changes in *E. coli*	13	51	41	12	9	38	21	4
Total # of changes in all genomes	92	357	252	88	169	522	363	40
Total # of changes that agree with a validated start	9	76	82	31	20	114	126	28
Total # of changes that disagree with a validated start	4	7	6	1	4	15	0	0

a *E = FP*/(*TP*+*FP*), where *TP* = number of true positives (second row), and *FP* = number of false positives (third row).

b Estimated by adding one additional false positive to obtain a nonzero value.

c *S = TP/GP*, where *TP = *number of true positives (second row), and *GP = *number of ground truth positives (first row).

### Projected impact on consistency of microbial ortholog sets

To estimate the broader impact of GMV, we calculated the increase in consistency for the medium and high diversity genome test sets with either 5 or 10 genomes and then applied these rates to 39 genera. For each test set we obtained the number of genes *m_k_* predicted by Prodigal for each genome *k* and selected the smallest number, *M* = min(*m*_1_, *m*_2_, …, *m_k_*). The value of *M* corresponds to the maximum possible number of ortholog sets for a genome set. Values of *M* are given in Table 5. Next, we calculated the ortholog set yield *Y* = *O*/*M*, where *O* is the actual number of ortholog sets obtained for each genome set (see first row of Table 1). The yield for medium diversity was *Y*≈1/2, and for high diversity *Y*≈1/3, roughly independent of the number of genomes in the set (Table 5). Finally we calculated the increase in consistency after running GMV, which ranged from *I* = 11.4% to *I* = 17.2%, calculated as a percentage of the number of actual ortholog sets (Table 5). To estimate the number of ortholog sets with increased consistency, *n_I_*, after running GMV, we used the equation
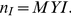
(4)


**Table 5:** Ortholog set yield calculated for medium and high diversity genome test sets.

	5 genomes		10 genomes	
	Medium	High	Medium	High
Maximum possible # ortholog sets, *M*	4282	4332	4151	3710
Ortholog set yield, *Y* a	57.1%	32.6%	51.3%	35.4%
Increase in consistency after applying GMV, *I* b	11.4%	14.4%	13.4%	17.2%

a Calculated as percentage of *M* using values from the first row of Table 1.

b Calculated as percentage of the number actual ortholog sets by subtracting the third from the fifth row of Table 1.

We identified suitable target genomes from a list of finished microbial genomes from the Integrated Microbial Genomes resource (http://img.jgi.doe.gov/cgi-bin/pub/main.cgi). We identified 39 genera, each containing a minimum number of 5 finished genomes, as likely targets for our method (Supplementary Table S2). We calculated *M* for each genus and obtained a conservative estimate of the total number of ortholog sets made consistent for each genus using Equation (4) with the lowest values *Y* = 0.326 and *I* = 0.114 from Table 5. The total estimated number of ortholog sets made consistent for 467 genomes was about 4,000.

Although the precise values of *Y* and *I* will differ depending on the genus, using the above values is reasonable for our conservative rough estimate. In Table 5 the value of *Y* only weakly varies with the number of genomes (e.g. *Y* declined only about 10% when the number of genomes doubled from 5 to 10), but is sensitive to sequence diversity. Sequence diversity does change depending on the genus; however, we visually inspected a bacterial phylogenetic tree (Supplementary Fig. S9) and found that the evolutionary divergence among the genus-level genome sets is similar to the medium and high diversity genome sets analyzed above, providing evidence that the value of *Y* is at least in the right ballpark. In addition, because Prodigal performs comparatively very well on *E. coli* [3], the value of *I* is likely a lower bound on the value that would apply to the 39 genera. For many of these genera, higher initial error rates in gene start site prediction would likely yield a larger number of ortholog sets that could be corrected, as we reported previously with *Burkholderia* genomes [7].

### Consistency of GenBank versus Prodigal maps

The impact of GMV estimated with Prodigal gene maps is likely to be conservative because Prodigal gene predictions (for orthologs) tend to be more consistent than extant Genbank data. By “Genbank data”, we mean the owner-approved or “curated” maps that are accessed by default in Genbank. As described previously [6], the percentage of ortholog sets with inconsistent start sites among 5 *Burkholderia* genomes (representing a medium diversity set) was 53% and 35%, respectively, based on Genbank maps and Prodigal maps. Similar results were obtained in the current study with ortholog sets from the comparable 5-genome, medium diversity *E. coli* test set (Table 6; Supplementary Table S3). In this test set, 2,289 ortholog sets were common to GenBank maps and Prodigal maps, enabling a direct comparison of consistency rates. Twice as many ortholog sets had inconsistent GenBank start sites (925, or 40.4%) as had inconsistent Prodigal start sites (455, or 19.9%). Prodigal made 60% (552) of the 925 GenBank ortholog sets consistent, and GMV made an additional 21% consistent. Together, Prodigal and GMV made 81% (746) of the GenBank ortholog sets consistent. This corresponds to an increase in consistency of *I* = 746/2289 = 0.326.

**Table 6:** Comparison of inconsistencies for Prodigal vs. GenBank or Glimmer3 start sites.

		5 genomes Medium Diversity	5 genomes High Diversity
Prodigal vs. GenBank	# of shared ortholog sets	2289	1234
	# of ortholog sets for which Prodigal starts were initially inconsistent	455	413
	# of ortholog sets for which GenBank starts were initially inconsistent	925	311
	# made consistent by Prodigal	552	50
	# made consistent by GMV	194	47
Prodigal vs. Glimmer3	# of shared ortholog sets	2427	1398
	# of ortholog sets for which Prodigal starts were initially inconsistent	532	566
	# of ortholog sets for which GenBank starts were initially inconsistent	869	767
	# made consistent by Prodigal	432	248
	# made consistent by GMV	193	155

The above conservative estimate suggests applying our pipeline could significantly increase consistency of GenBank gene maps, with Prodigal accounting for ¾ and GMV accounting for ¼ of the total impact. We also obtained an alternative, less conservative estimate of the impact by modifying the GMV algorithm to preferentially use the gene calls already in GenBank as opposed to new Prodigal gene calls. In the modified algorithm, if the GenBank start sites already coincide in a multiple sequence alignment, or if a majority of these start sites do not align, nothing is done. Otherwise, if a majority of the GenBank start sites coincide, an alternative, consistent Prodigal start site is sought in the minority genomes. If one is found, then in the minority genomes the GenBank start sites are replaced with the consistent Prodigal start sites. Applying this algorithm to the same 2,289 ortholog sets that were common to GenBank maps and Prodigal maps in the 5-genome, medium diversity *E. coli* test set made 78% (717) of the 925 inconsistent GenBank ortholog sets consistent. This number is comparable to the 81% made consistent by first substituting all of the GenBank start sites with Prodigal start sites, and then applying GMV; however, in this alternative mode GMV was responsible for all of the changes as opposed to ¼ of the changes in the original mode. Using the method described for the Prodigal projection above, we project that running GMV in this alternative mode on 467 currently sequenced microbial genomes (Supplementary Table S2) would make more than 10,000 ortholog sets consistent. Unfortunately, although GMV used in this alternative mode does improve consistency, because the GenBank gene maps for *E. coli* have already been modified to account for the 871 experimentally validated start sites, we cannot say whether such an application of GMV increases gene map accuracy. Therefore we have no good basis on which to recommend that GMV be used in this alternative mode, and we adhere to the more conservative projection that GMV would resolve about 4,000 inconsistencies in Prodigal gene maps, as estimated in the previous section.

### Projected impact on accuracy of microbial gene maps

To project the broader impact of the GMV method on the accuracy of Prodigal gene maps, we first calculated a correction rate for the medium and high diversity genome test sets with either 5 or 10 genomes and then applied this rate to 39 suitable genera. The correction rate *R* per genome was calculated as
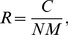
(5)where *M* is the maximum number of possible ortholog sets as defined in the previous section, *C* is the total number of changes in the set from Table 4, and *N* is the number of genomes in the set. Correction rates, *R*, for 5-genome test sets with medium and high diversity were 1.7% and 1.2% respectively, and were 1.2% and 1% for corresponding 10-genome test sets. The entire range was therefore 1% to 1.7%. To obtain the projected impact on accuracy, we used the same set of 39 genera (Supplementary Table S2) that were used to estimate the impact on consistency. With correction rates of 1% or 1.7%, the total estimated number of corrections for 467 genomes was 13,700 and 23,300, respectively. The estimated rate of erroneous corrections varied widely. The error rates calculated from Table 4 were 8.4% and 6.8% for 5-genome test sets of medium and high diversity, respectively, and 12% and 0.8% for 10-genome test sets of medium and high diversity. With the worst-case scenario (12% error rate), we project GMV to yield more than 10,000 valid gene start corrections in 467 microbial genomes.

The projection above applies to Prodigal gene maps; an assessment for existing Genbank gene maps is also desired. Accuracy can be directly measured for organisms, like *E. coli*, that have a gene map and a set of experimentally validated gene start sites. However, the Genbank gene map for *E. coli* has already been revised to include the 871 experimentally validated gene start sites, and therefore the accuracy of the map for these genes cannot be improved further. We must therefore estimate the impact based on the following logic: 1) the vast majority of Genbank maps are inferior in quality compared to the *E. coli* map, which has benefited from a rigorous community annotation effort [11]; 2) Genbank gene maps have as much as twice as many inconsistencies as Prodigal gene maps; and 3) we have directly measured the impact of GMV on the accuracy of Prodigal gene maps. Using the 12% error rate from the high diversity, 10-genome test set as a worst-case scenario for erroneous corrections, more than 20,000 valid corrections are projected for Genbank gene maps for 467 microbial genomes.

It is conceivable that the impact will be lower for new gene maps obtained from recent improvements in annotation pipelines. Newer maps may include information from servers such as MaGe [12], RAST [8], or GenePRIMP [9] that use comparative genomics methods for genome annotation, including leveraging information from experimentally validated gene starts. Given the evolving quality of newer gene maps, the true value of the GMV method in correcting errors in GenBank genomes will depend on accumulation of data from a broader set of users.

### Significance of the GMV algorithm in light of other methods

Ours is one of several approaches to leveraging multiple genomes for improving gene predictions. Numerous methods have used conservation patterns in pairwise sequence alignments to distinguish coding from non-coding regions in eukaryotic (SLAM [13], SGP2 [14], TWINSCAN [15], [16], [17], Guigó et al. [18]) or prokaryotic genomes (Walker et al. [19],). The use of more than two sequences to improve prediction of gene boundaries is a more recent addition.

RAST [8] and GenePRIMP [9] both use homologs identified via BLAST [20] to refine assignment of gene starts to new genomes. However, the decision rules and implementation details for revising gene start sites using these methods were not documented in detail. For example, the total number of orthologs that are used for comparison, selection of diversity among orthologs (when a spectrum of diversity is available), and the definition of consistency for these methods are unclear. These methods might make an effort to minimize 5′ length differences among orthologs, which does enforce a kind of consistency, in the spirit of GMV. However, our *Burkholderia* study [7] appears to be the first example of a strict rule to enforce consistency of start sites in a multiple sequence alignment. Another important distinction between GMV and these methods is their use of “old” gene predictions as reference material (obtained when acquiring homologs from databases with archived, error-prone information) versus contemporary gene predictions. RAST and GenePRIMP both exploit archived material, in which the extent of errors is unknown. As shown above for the 5-genome, medium diversity genome set, our GMV algorithm can, in principle, exploit older gene maps; however, we can only recommend exploiting new gene predictions. As discussed above, new predictions are expected to be more accurate and, compared to gene maps already deposited in the databases, the lack of manual adjustment of these predictions enabled them to be used to rigorously assess the performance of GMV using experimentally validated genes. The automation of this pipeline provides a facile means to periodically upgrade gene maps for collections of older genomes, as well as improving gene start site predictions for new genomes.

N-SCAN [21] and CONTRAST [22] can produce gene calls using information from more than two genomes. N-SCAN leverages a phylogenetic model to improve gene prediction. CONTRAST improved on N-SCAN by doing away with the phylogenetic model in favor of a machine learning approach. It is notable that, with the exception of CONTRAST, prior comparative genomics approaches were unable to demonstrate improved gene start site predictions beyond adding a second genome, much less more [23]. CONTRAST demonstrated small improvements as the number of genomes was increased to five [22]. The fact that the performance of GMV improved when the number of genomes was increased from five to ten makes it unique among comparative genomics methods.

A shared feature of prior approaches is that the multiple genomes are input at the front end and are used to develop a tightly integrated gene prediction model. By contrast, the GMV algorithm is run as a post-processing step. The main disadvantage of this is the additional compute time required to refine gene calls: running GMV on a 5-genome set takes about ½ a day on a single processor machine. The compute time is limited by the BLAST step, which scales like the number of genomes squared; however, the speed of the BLAST step (and all other steps of the pipeline) can be substantially improved by parallel processing. A major advantage in implementing GMV is that it can be coupled to any gene prediction software so long as a list of alternative start sites is provided. Aside from the great flexibility it provides in applications, the modular nature of GMV allowed us to treat it as an error correction method, enabling a well-controlled means of evaluating its performance.

### Conclusions

The GMV algorithm dramatically decreases inconsistencies in the location of predicted gene start sites, and is projected to eliminate thousands of inconsistencies in currently sequenced microbial genomes, facilitating comparative genomics studies. At the same time, it is capable of correcting hundreds of errors in sets of 5–10 genomes and is potentially capable of correcting more than 10,000 errors in microbial gene maps. Moreover, GMV provides a straightforward solution to the challenging problem of improving gene start site predictions using more than two genomes. Overall, GMV is a simple and logical solution that resolves inconsistencies and increases the accuracy of gene maps.

## Methods

### Genome sets

Genome sets were selected with the aid of a bacterial phylogenetic tree (Benjamin McMahon, personal communication). The tree was derived by aligning the concatenated amino acid sequences of the β and β′ subunits of RNA polymerase from over 400 bacterial genomes. The 400 bacterial genomes were downloaded from NCBI (completed) and JGI (draft) in June of 2009. The amino acid sequences of the beta and beta-prime subunits of the RNA polymerase were extracted from each genome and concatenated. An initial multiple sequence alignment was calculated using MUSCLE [24], followed by iterative manual curation of the alignment with BioEdit (http://www.mbio.ncsu.edu/bioedit/bioedit.html) based on the known three-dimensional structure, and tree building with a maximum likelihood method employing a minimal model of protein functional pressure (RIND [25] and WEIGHBOR [26]). The phylogenetic tree was calculated from the aligned sequences. The root of the tree was placed at the long branch connecting gram-positive and gram-negative bacteria, in accord with current understanding of bacterial evolution [27]. The resulting tree (Supplementary 9) compares well to those in the literature [28] and with 16S rRNA-based trees; it disagrees with the less-detailed NCBI taxonomy (where available) in only a handful of cases.

The genome sets used for testing GMV are listed in Supplementary Table S2. Sets of 10-genomes were selected to represent low, medium, and high, and very high levels of diversity. The low diversity sets consist of randomly selected substrains of *E. coli*. The medium diversity sets were selected with the aid of the phylogenetic tree (Supplementary Fig. S9) to approximately span a maximum evolutionary distance similar to that spanned by the *Burkholderia* genus, which was the subject of our previous analysis of gene start consistency [7]. The genomes selected for the medium diversity sets cover a portion of the *Enterobacteriaceae* family. The high diversity datasets were selected to achieve approximately a twofold increase in the maximum evolutionary distance over the medium diversity datasets and cover a larger portion of the *Enterobacteriaceae* family. The very high diversity datasets were selected to increase the evolutionary distance by another factor of two. The very high diversity datasets include genomes from two families of Gamma Proteobacteria: *Enterobacteriaceae* and *Pasteurellaceae*. After selecting the 10-genome sets, subsets of 5 genomes were down-selected for each diversity level.

**Figure 4:**
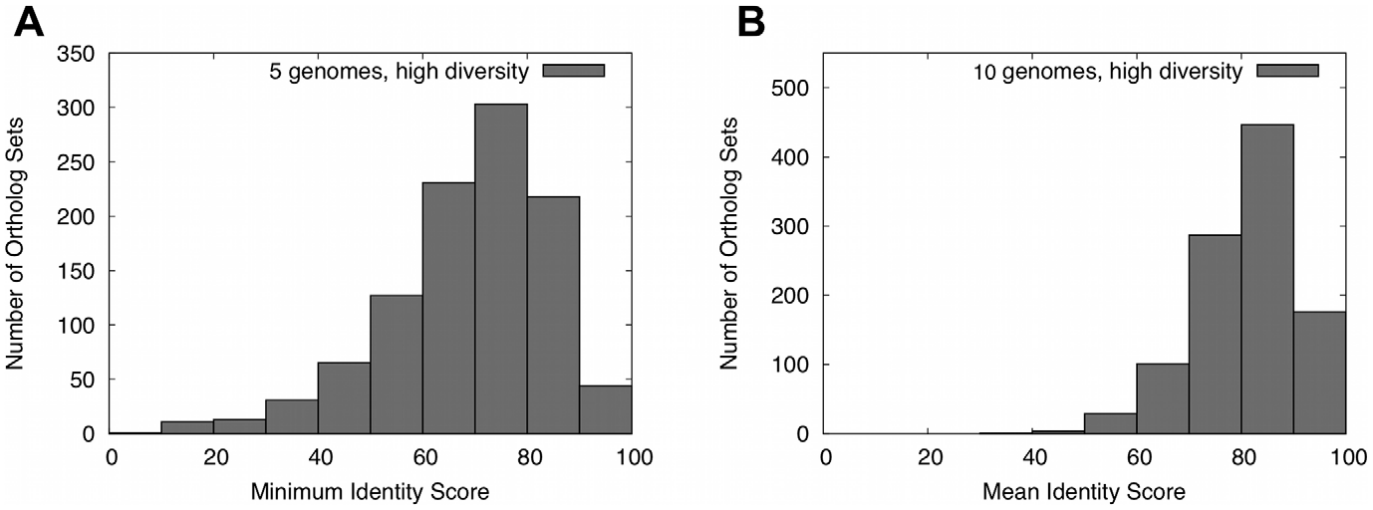
Sequence identity statistics for the high diversity ortholog sets. A) 5-genome set; B) 10-genome test set.

Table 7 summarizes the diversity in each of the 8 test sets using the median of the minimum sequence identity in each set. Fig. 4 illustrates more detailed statistics on the sequence identity in the high diversity genome sets, which yielded the best performance for the GMV algorithm. Supplementary Figs. S_1_–S_8_ provide this information for all genome sets. Supplementary Table S1 lists the genomes in all genome sets. (Note that the low diversity genome test sets include several strains of *E. coli* genomes; in this paper, we refer to *E. Coli* K-12 MG1655, the reference genome, as *E. coli*.)

**Table 7:** Median sequence identities among orthologs from all genome test sets.

# Genomes, Diversity	Median Sequence Identity^a^
5, Low	99.3%
5, Medium	85.2%
5, High	71.6%
5, Very High	64.4%
10, Low	98.8%
10, Medium	82.5%
10, High	69.5%
10, Very High	51.3%

a The sequence identity used is the minimum value among all gene pairs in each ortholog set. The percentage value is normalized using sequence length information (Methods).

### GMV algorithm

The GMV algorithm was implemented in an automated pipeline to predict consistent start sites, illustrated in Fig. 1. The software is distributed freely under a New BSD license and is available at http://code.google.com/p/gmv/. The input is a set of similar genomes whose start sites are to be predicted. These genomes are provided in FASTA format to the GMV algorithm. The different steps in the pipeline involve four different software programs, each of which is automatically activated:

#### Step A

In this step, gene predictions are made for each genome in the set using Prodigal (we used version 1.10 here, which is no longer available; the version we distribute uses versions 2.00–2.50) [3]. Like most gene finding programs, Prodigal selects a single best start site but also evaluates other potential start sites for each gene, computing a quality score for each start site that it proposes. The GMV algorithm uses the alternative start sites in the subsequent steps of the pipeline.

#### Step B

In this step, alternative start sites for each gene in each genome are obtained from the Prodigal output files.

#### Step C

In this step, gene predictions from Step B are used to derive ortholog sets by a pan-reciprocal best hit approach using BLASTP (version 2.2.20) with default settings [20]. First, BLASTP is used to obtain sequence identity for all pairs of proteins corresponding to the genes predicted by Prodigal. The sequence identity score computed by BLASTP is normalized by multiplying it by the number of aligned bases and dividing it by the number of bases in the longer of the two compared sequences. Sets of orthologous genes that include a single pan-reciprocal best BLASTP match for each genome are identified; matches are ranked by the normalized sequence identity computed above. Each ortholog set contains exactly one representative from each genome in the set.

#### Step D

Multiple sequence alignment is performed for each ortholog set using MUSCLE (version 3.7) with default settings [24]. The sequence of each gene in the alignment includes the 250 bp DNA sequence upstream of the earliest of the possible starts. Nucleotide sequences are used to construct multiple sequence alignments.

#### Step E

This is the final step in the GMV pipeline and it involves prediction of consistent start sites. If the positions of all of the original start sites coincide in the multiple sequence alignment, the predictions are accepted as is. Otherwise, look for a position where the original start sites coincide for a majority of genomes, and where an alternative start site coincides in each of the remaining genomes. Use the alternative sites as modified predictions for the remaining genomes. If there is no consistent start site that obeys the majority rule, flag the prediction as inconsistent.

It is important to note that the GMV pipeline is not restricted to using Prodigal for gene prediction and MUSCLE for multiple sequence alignment. GMV can be made to work with any gene prediction software that can output alternative start sites in Step A. The current requirements for input to GMV is described in the manual included in the package, distributed at http://code.google.com/p/gmv/. Similarly, it is possible to use any multiple sequence alignment software that can handle nucleotide data in Step D of the pipeline. The entire pipeline can be run automatically, without any manual intervention. It is also possible to run each step of the pipeline separately, if necessary.

### Software

The GMV algorithm pipeline was developed using Java (JDK 1.6) and Perl 5.8. The software has been tested on both Linux and MacOS X operating systems. Software is available under the New BSD open source license and is freely available at http://code.google.com/p/gmv.

## Supporting Information

Figure S1 Histogram of mean (top) and minimum (bottom) identity score between genes in ortholog sets derived from the low diversity, 5 genome set.
(PDF)

Figure S2 Histogram of mean (top) and minimum (bottom) identity score between genes in ortholog sets derived from the medium diversity, 5 genome set.
(PDF)

Figure S3 Histogram of mean (top) and minimum (bottom) identity score between genes in ortholog sets derived from the high diversity, 5 genome set.
(PDF)

Figure S4 Histogram of mean (top) and minimum (bottom) identity score between genes in ortholog sets derived from the very high diversity, 5 genome set.
(PDF)

Figure S5 Histogram of mean (top) and minimum (bottom) identity score between genes in ortholog sets derived from the low diversity, 10 genome set.
(PDF)

Figure S6 Histogram of mean (top) and minimum (bottom) identity score between genes in ortholog sets derived from the medium diversity, 10 genome set.
(PDF)

Figure S7 Histogram of mean (top) and minimum (bottom) identity score between genes in ortholog sets derived from the high diversity, 10 genome set.
(PDF)

Figure S8 Histogram of mean (top) and minimum (bottom) identity score between genes in ortholog sets derived from the very high diversity, 10 genome set.
(PDF)

Figure S9 Bacterial phylogenetic tree. The tree is based on aligning the beta and beta-prime subunits of the RNA polymerase and was generated using a maximum likelihood method [25], [26]. The root of the tree is at the left, on the long branch connecting gram-positive and gram-negative bacteria. The lengths of horizontal lines correspond to a measure of evolutionary distance.
(PDF)

Table S1 List of genomes in each genome set. The FASTA files were downloaded June–July 2010.
(PDF)

Table S2 List of genomes used to estimate projected impact of GMV on consistency and error rates in gene predictions. The 467 genomes were organized into 39 genera for the estimate. The genome list was obtained from the Integrated Microbial Genomes resource at the DOE Joint Genome Institute (http://img.jgi.doe.gov/cgi-bin/pub/main.cgi) in September 2010.
(PDF)

Table S3 Source files for GenBank default and Glimmer3 gene start sites for 5 genome sets of medium and high diversity.
(PDF)

## References

[1] Delcher AL, Bratke KA, Powers EC, Salzberg SL (2007). Identifying bacterial genes and endosymbiont DNA with Glimmer.. Bioinformatics.

[2] Besemer J, Borodovsky M (2005). GeneMark: web software for gene finding in prokaryotes, eukaryotes and viruses.. Nucleic Acids Res.

[3] Hyatt D, Chen GL, Locascio PF, Land ML, Larimer FW (2010). Prodigal: prokaryotic gene recognition and translation initiation site identification.. BMC Bioinformatics.

[4] Dai M, Wang P, Boyd AD, Kostov G, Athey B (2005). Evolving gene/transcript definitions significantly alter the interpretation of GeneChip data.. Nucleic Acids Res.

[5] Poptsova MS, Gogarten JP (2010). Using comparative genome analysis to identify problems in annotated microbial genomes.. Microbiology.

[6] Pallejà A, Harrington ED, Bork P (2008). Large gene overlaps in prokaryotic genomes: result of functional constraints or mispredictions?. BMC Genomics.

[7] Dunbar J, Cohn JD, Wall ME (2011). Consistency of gene starts among *Burkholderia* genomes.. BMC Bioinformatics.

[8] Aziz RK, Bartels D, Best AA, DeJongh M, Disz T (2008). The RAST Server: rapid annotations using subsystems technology.. BMC Genomics.

[9] Pati A, Ivanova NN, Mikhailova N, Ovchinnikova G, Hooper SD (2010). GenePRIMP: a gene prediction improvement pipeline for prokaryotic genomes.. Nat Methods.

[10] Rudd KE (2000). EcoGene: a genome sequence database for *Escherichia coli* K-12.. Nucleic Acids Res.

[11] Riley M, Abe T, Arnaud MB, Berlyn MK, Blattner FR (2006). *Escherichia coli* K-12: a cooperatively developed annotation snapshot–2005.. Nucleic Acids Res.

[12] Vallenet D, Labarre L, Rouy Z, Barbe V, Bocs S (2006). MaGe: a microbial genome annotation system supported by synteny results.. Nucleic Acids Res.

[13] Alexandersson M, Cawley S, Pachter L (2003). SLAM: cross-species gene finding and alignment with a generalized pair hidden Markov model.. Genome Res.

[14] Parra G, Agarwal P, Abril JF, Wiehe T, Fickett JW (2003). Comparative gene prediction in human and mouse.. Genome Res.

[15] Flicek P, Keibler E, Hu P, Korf I, Brent MR (2003). Leveraging the mouse genome for gene prediction in human: from whole-genome shotgun reads to a global synteny map.. Genome Res.

[16] Korf I, Flicek P, Duan D, Brent MR (2001). Integrating genomic homology into gene structure prediction.. Bioinformatics.

[17] Tenney AE, Brown RH, Vaske C, Lodge JK, Doering TL (2004). Gene prediction and verification in a compact genome with numerous small introns.. Genome Res.

[18] Guigó R, Dermitzakis ET, Agarwal P, Ponting CP, Parra G (2003). Comparison of mouse and human genomes followed by experimental verification yields an estimated 1,019 additional genes.. Proc Natl Acad Sci U S A.

[19] Walker M, Pavlovic V, Kasif S (2002). A comparative genomic method for computational identification of prokaryotic translation initiation sites.. Nucleic Acids Res.

[20] Altschul SF, Gish W, Miller W, Myers EW, Lipman DJ (1990). Basic local alignment search tool.. J Mol Biol.

[21] Gross SS, Brent MR (2006). Using multiple alignments to improve gene prediction.. J Comput Biol.

[22] Gross SS, Do CB, Sirota M, Batzoglou S (2007). CONTRAST: a discriminative, phylogeny-free approach to multiple informant de novo gene prediction.. Genome Biol.

[23] Brent MR (2008). Steady progress and recent breakthroughs in the accuracy of automated genome annotation.. Nat Rev Genet.

[24] Edgar RC (2004). MUSCLE: multiple sequence alignment with high accuracy and high throughput.. Nucleic Acids Res.

[25] Bruno WJ (1996). Modeling residue usage in aligned protein sequences via maximum likelihood.. Mol Biol Evol.

[26] Bruno WJ, Socci ND, Halpern AL (2000). Weighted neighbor joining: a likelihood-based approach to distance-based phylogeny reconstruction.. Mol Biol Evol.

[27] Skophammer RG, Servin JA, Herbold CW, Lake JA (2007). Evidence for a gram-positive, eubacterial root of the tree of life.. Mol Biol Evol.

[28] Herlemann DP, Geissinger O, Ikeda-Ohtsubo W, Kunin V, Sun H (2009). Genomic analysis of “*Elusimicrobium minutum*,” the first cultivated representative of the phylum “*Elusimicrobia*” (formerly termite group 1).. Appl Environ Microbiol.

