# Supplementary material for: Genome Majority Vote Improves Gene Predictions
Source: PLoS Comput Biol. 2011 Nov 17;7(11):e1002284. doi: 10.1371/journal.pcbi.1002284 (PMC3219611; doi:10.1371/journal.pcbi.1002284)
Supplement: Figure S2 — Histogram of mean (top) and minimum (bottom) identity score between genes in ortholog sets derived from the medium diversity, 5 genome set. (PDF) [file pcbi.1002284.s002.pdf]

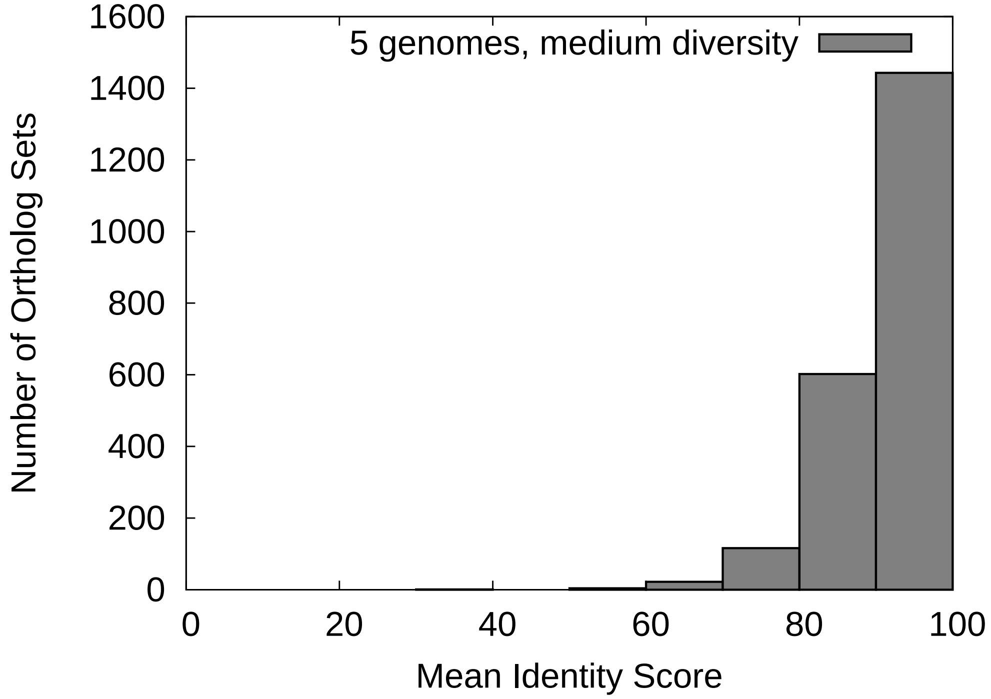

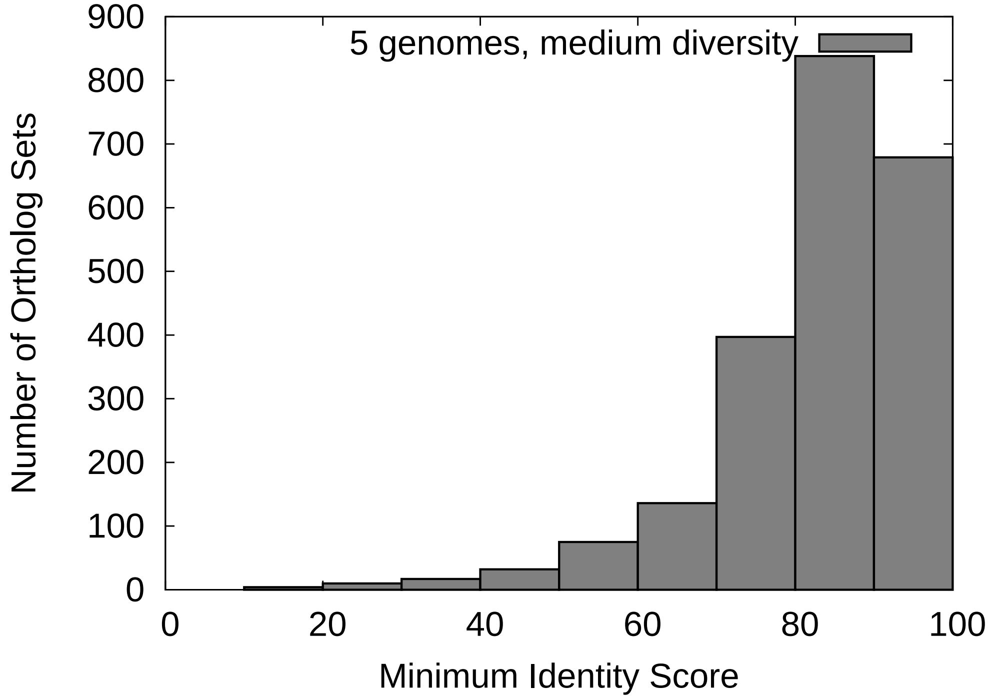

Figure S2: Histogram of mean (top) and minimum (bottom) identity score between genes in ortholog sets derived from the medium diversity, 5 genome set.
