# Supplementary figures and images for: Genome Majority Vote Improves Gene Predictions
Source: PLoS Comput Biol. 2011 Nov 17;7(11):e1002284. doi: 10.1371/journal.pcbi.1002284 (PMC3219611; doi:10.1371/journal.pcbi.1002284)

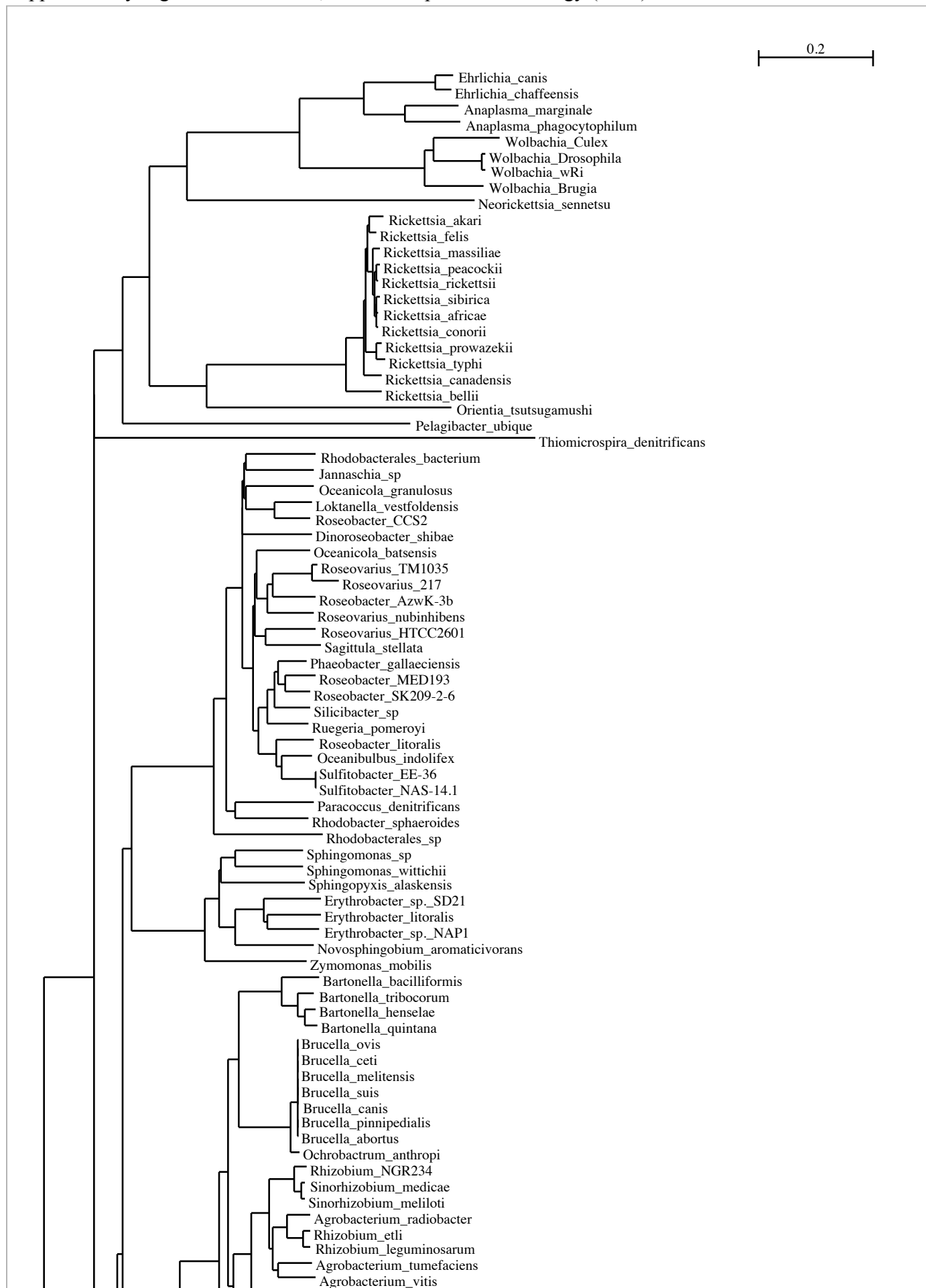

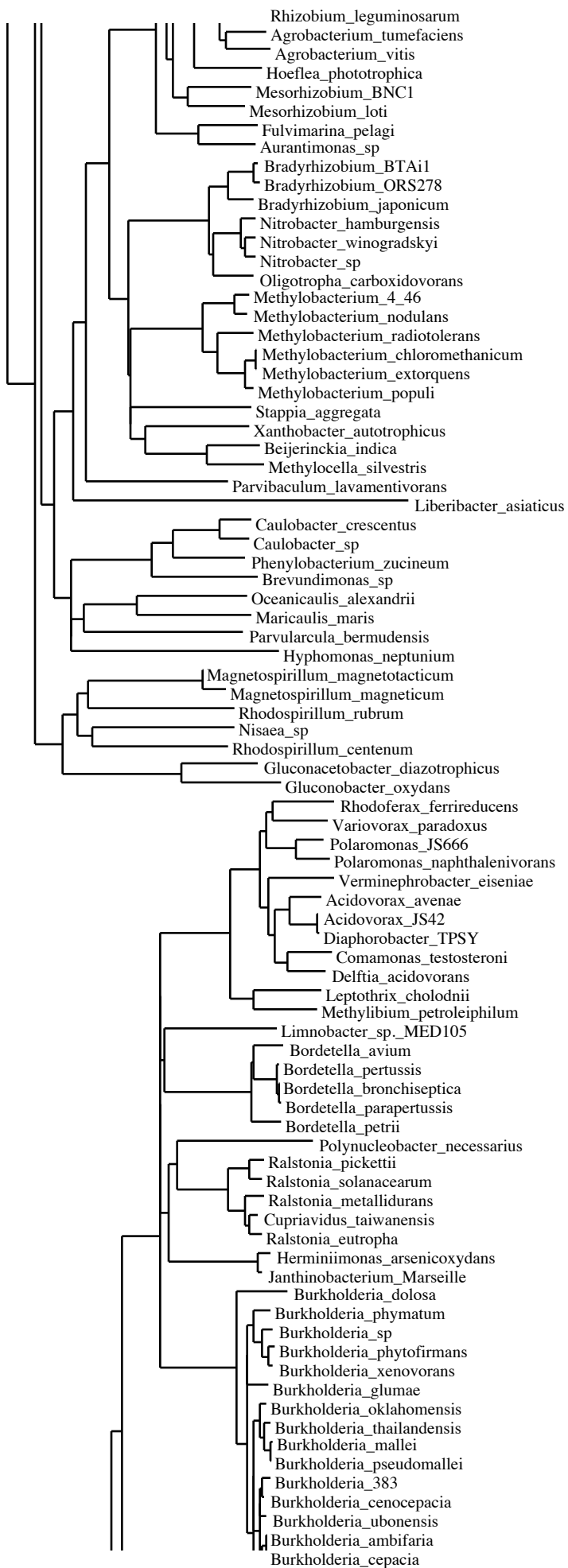

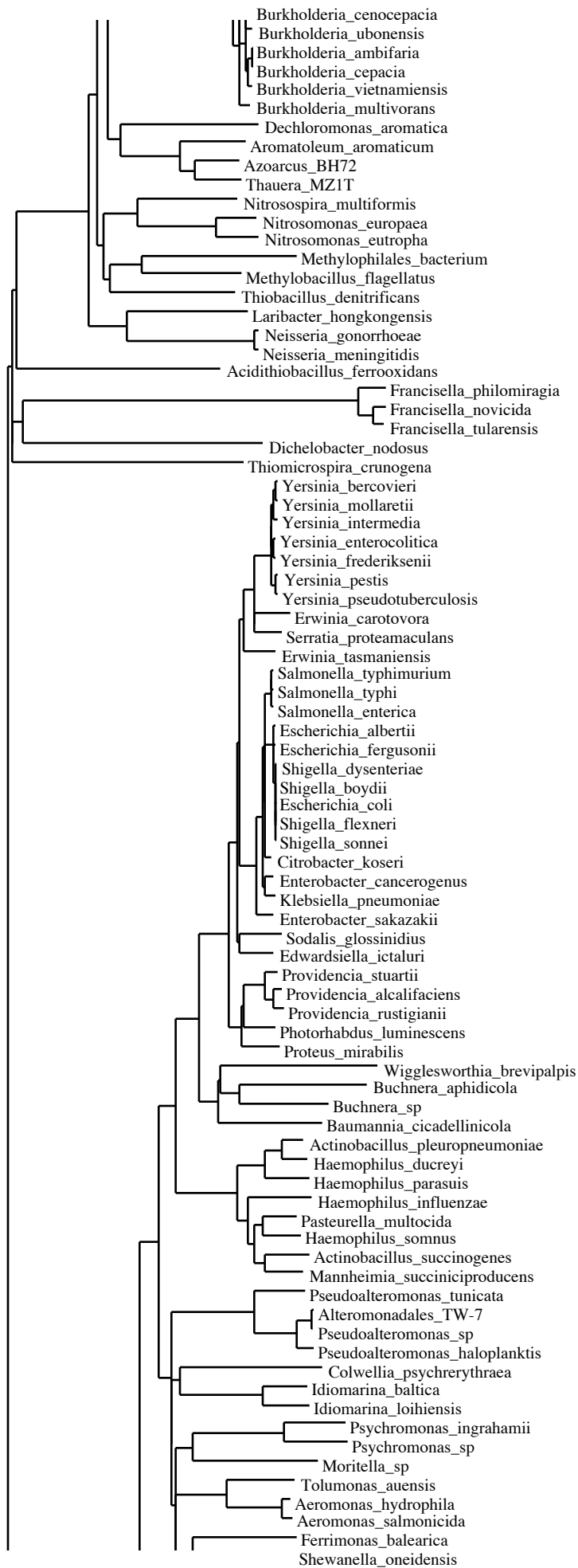

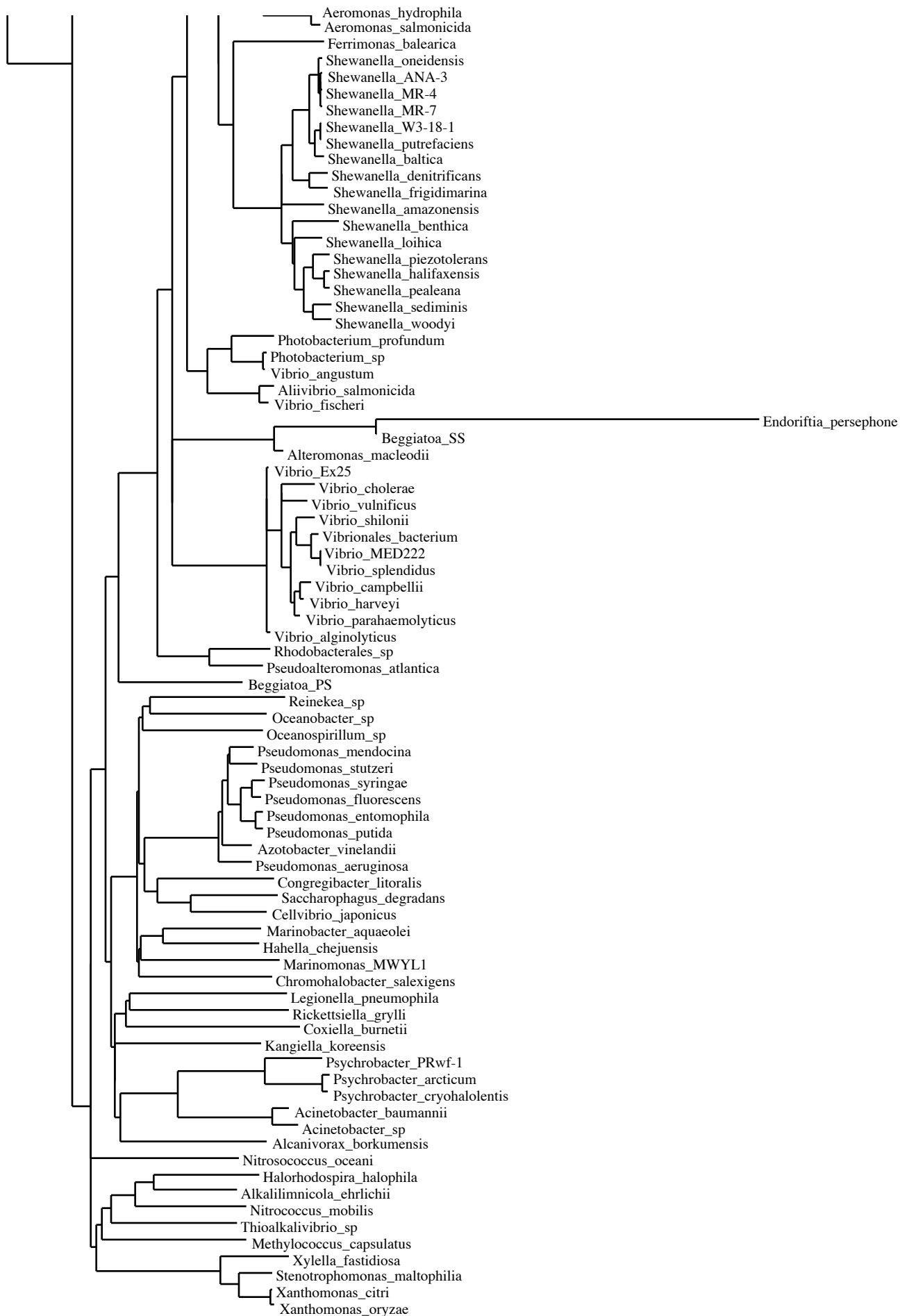

Supplement: Figure S9 — Bacterial phylogenetic tree. The tree is based on aligning the beta and beta-prime subunits of the RNA polymerase and was generated using a maximum likelihood method [25], [26]. The root of the tree is at the left, on the long branch connecting gram-positive and gram-negative bacteria. The lengths of horizontal lines correspond to a measure of evolutionary distance. (PDF) [file pcbi.1002284.s009.pdf]
