# Supplementary material for: Genome Majority Vote Improves Gene Predictions
Source: PLoS Comput Biol. 2011 Nov 17;7(11):e1002284. doi: 10.1371/journal.pcbi.1002284 (PMC3219611; doi:10.1371/journal.pcbi.1002284)
Supplement: Table S1 — List of genomes in each genome set. The FASTA files were downloaded June–July 2010. (PDF) [file pcbi.1002284.s010.pdf]

Supplementary Table S1 for M.E. Wall *et al*., Genome majority vote improves gene predictions, *PLoS Computational Biology* (2011).

Genome Set		Genome	FASTA Files
5 genomes	Low diversity	Escherichia coli 55989	NC_011748.fna
		Escherichia coli E24377A	NC_009786.fna,NC_009788.fna,NC_009790.fna,NC_009801.fna, NC_009791.fna,NC_009789.fna,NC_009787.fna
		Escherichia coli IAI1	NC_011741.fna
		Escherichia coli str. K-12 substr. MG1655	NC_000913.fna
		Escherichia coli O157:H7 str. Sakai	NC_002127.fna,NC_002695.fna,NC_002128.fna
	Medium diversity	Citrobacter koseri ATCC BAA-895	NC_009792.fna,NC_009794.fna,NC_009793.fna
		Escherichia coli str. K-12 substr. MG1655	NC_000913.fna
		Klebsiella pneumoniae 342	NC_011281.fna,NC_011283.fna,NC_011282.fna
		Salmonella enterica subsp. arizonae serovar 62:z4	NC_010067.fna
		Shigella flexneri 2a str. 301	NC_004337.fna,NC_004851.fna
	High diversity	Escherichia coli str. K-12 substr. MG1655	NC_000913.fna
		Erwinia carotovora subsp. atroseptica SCRI1043	NC_004547.fna
		Klebsiella pneumoniae 342	NC_011281.fna,NC_011283.fna,NC_011282.fna
		Photorhabdus luminescens subsp. laumondii TTO1	NC_005126.fna
		Sodalis glossinidius str. 'morsitans'	NC_007712.fna,NC_007713.fna,NC_007714.fna,NC_007715.fna
	Very High diversity	Actinobacillus succinogenes 130Z	NC_009655.fna
		Escherichia coli str. K-12 substr. MG1655	NC_000913.fna
		Haemophilus influenzae Rd KW20	NC_000907.fna
		Photorhabdus luminescens subsp. laumondii TTO1	NC_005126.fna
		Yersinia pestis Nepal516	NC_008118.fna,NC_008119.fna,NC_008149.fna
10 genomes	Low diversity	Escherichia coli 536	NC_008253.fna
		Escherichia coli 55989	NC_011748.fna
		Escherichia coli APEC O1	NC_008563.fna,NC_009837.fna,NC_009838.fna
		Escherichia coli BW2952	NC_012759.fna
		Escherichia coli E24377A	NC_009786.fna,NC_009788.fna,NC_009790.fna,NC_009801.fna, NC_009791.fna,NC_009789.fna,NC_009787.fna
		Escherichia coli HS	NC_009800.fna
		Escherichia coli IAI1	NC_011741.fna
		Escherichia coli str. K-12 substr. MG1655	NC_000913.fna
		Escherichia coli O157:H7 str. Sakai	NC_002127.fna,NC_002695.fna,NC_002128.fna
		Escherichia coli SE11	NC_011407.fna,NC_011411.fna,NC_011415.fna,NC_011419.fna, NC_011416.fna,NC_011413.fna,NC_011408.fna
	Medium diversity	Citrobacter koseri ATCC BAA-895	NC_009792.fna,NC_009793.fna,NC_009794.fna
		Escherichia coli str. K-12 substr. MG1655	NC_000913.fna
		Enterobacter sakazakii ATCC BAA-894	NC_009778.fna,NC_009780.fna,NC_009779.fna
		Escherichia fergusonii ATCC 35469	NC_011740.fna,NC_011743.fna
		Klebsiella pneumoniae 342	NC_011281.fna,NC_011282.fna,NC_011283.fna
		Salmonella enterica subsp. arizonae serovar 62:z4	NC_010067.fna
		Salmonella enterica subsp. enterica serovar Typhi str. CT18	NC_003198.fna,NC_003384.fna,NC_003385.fna
		Salmonella typhimurium LT2	NC_003197.fna,NC_003277.fna
		Shigella dysenteriae Sd197	NC_007606.fna,NC_009344.fna,NC_007607.fna
		Shigella flexneri 2a str. 301	NC_004337.fna,NC_004851.fna
	High diversity	Citrobacter koseri ATCC BAA-895	NC_009792.fna,NC_009794.fna,NC_009793.fna
		Escherichia coli str. K-12 substr. MG1655	NC_000913.fna
		Erwinia carotovora subsp. atroseptica SCRI1043	NC_004547.fna
		Klebsiella pneumoniae 342	NC_011281.fna,NC_011283.fna,NC_011282.fna
		Photorhabdus luminescens subsp. laumondii TTO1	NC_005126.fna
		Proteus mirabilis H14320	NC_010554.fna,NC_010555.fna
		Salmonella enterica subsp. arizonae serovar 62:z4	NC_010067.fna
		Shigella flexneri 2a str. 301	NC_004337.fna,NC_004851.fna
		Sodalis glossinidius str. 'morsitans'	NC_007712.fna,NC_007713.fna,NC_007715.fna,NC_007714.fna
		Yersinia pestis Nepal516	NC_008118.fna,NC_008149.fna,NC_008119.fna
	Very High diversity	Actinobacillus pleuropneumoniae L20	NC_009053.fna
		Actinobacillus succinogenes 130Z	NC_009655.fna
		Buchnera aphidicola str. Bp (Baizongia pistaciae)	NC_004545.fna,NC_004555.fna
		Escherichia coli str. K-12 substr. MG1655	NC_000913.fna
		Erwinia carotovora subsp. atroseptica SCRI1043	NC_004547.fna
		Haemophilus influenzae Rd KW20	NC_000907.fna
		Klebsiella pneumoniae 342	NC_011281.fna,NC_011283.fna,NC_011282.fna
		Pasteurella multocida subsp. multocida str. Pm70	NC_002663.fna
		Photorhabdus luminescens subsp. laumondii TTO1	NC_005126.fna
		Yersinia pestis Nepal516	NC_008118.fna,NC_008119.fna,NC_008149.fna
