# Supplementary material for: Genome Majority Vote Improves Gene Predictions
Source: PLoS Comput Biol. 2011 Nov 17;7(11):e1002284. doi: 10.1371/journal.pcbi.1002284 (PMC3219611; doi:10.1371/journal.pcbi.1002284)
Supplement: Table S2 — List of genomes used to estimate projected impact of GMV on consistency and error rates in gene predictions. The 467 genomes were organized into 39 genera for the estimate. The genome list was obtained from the Integrated Microbial Genomes resource at the DOE Joint Genome Institute (http://img.jgi.doe.gov/cgi-bin/pub/main.cgi) in September 2010. (PDF) [file pcbi.1002284.s011.pdf]

Supplementary Table S2 for M.E. Wall *et al.*, Genome majority vote improves gene predictions, *PLoS Computational Biology* (2011).

Genome #	taxon_oid	Genome Name	Gene Count
1	643348502	Acinetobacter baumannii AB0057	3892
2	643348503	Acinetobacter baumannii AB307-0294	3542
3	642555102	Acinetobacter baumannii ACICU	3841
4	640069301	Acinetobacter baumannii ATCC 17978	3464
5	641522602	Acinetobacter baumannii AYE	3813
6	641522603	Acinetobacter baumannii SDF	3062
7	637000002	Acinetobacter sp. ADP1	3433
8	640753005	Bacillus amyloliquefaciens FZB42	3814
9	643692005	Bacillus anthracis A0248	5418
10	637000014	Bacillus anthracis Ames	5545
11	637000013	Bacillus anthracis Ames Ancestor	5853
12	643692006	Bacillus anthracis CDC 684	6031
13	637000015	Bacillus anthracis Sterne	5521
14	643692007	Bacillus cereus 03BB102	5767
15	643348510	Bacillus cereus AH187	5903
16	643348511	Bacillus cereus AH820	5941
17	637000016	Bacillus cereus ATCC 10987	6126
18	637000017	Bacillus cereus ATCC 14579	5513
19	643348512	Bacillus cereus B4264	5557
20	637000018	Bacillus cereus E33L	5886
21	643348513	Bacillus cereus G9842	5994
22	643348514	Bacillus cereus Q1	5621
23	640753006	Bacillus cereus cytotoxis NVH 391-98	4250
24	637000019	Bacillus clausii KSM-K16	4261
25	637000020	Bacillus halodurans C-125	4239
26	639279303	Bacillus licheniformis ATCC 14580 (Goettingen)	4356
27	639279304	Bacillus licheniformis ATCC 14580 (Novozymes)	4420
28	646311908	Bacillus pseudofirmus OF4	4434
29	640753007	Bacillus pumilus SAFR-032	3823
30	646311909	Bacillus subtilis subsp. subtilis str. 168	4354
31	639633008	Bacillus thuringiensis Al Hakam	5050
32	637000023	Bacillus thuringiensis sv konkukian 97-27	5452
33	641228477	Bacillus weihenstephanensis KBAB4	5983
34	639633010	Bifidobacterium adolescentis ATCC 15703	1709
35	643348515	Bifidobacterium animalis lactis AD011	1587
36	644736329	Bifidobacterium animalis subsp. lactis Bl-04	1631
37	644736330	Bifidobacterium animalis subsp. lactis DSM 10140	1629
38	646311910	Bifidobacterium dentium Bd1	2197
39	642555107	Bifidobacterium longum DJO10A	2074
40	637000031	Bifidobacterium longum NCC2705	1805

41	643348516	Bifidobacterium longum infantis ATCC 15697	2577
42	639633011	Borrelia afzelii PKo	1258
43	637000036	Borrelia burgdorferi B31	1690
44	643348517	Borrelia burgdorferi ZS7	1275
45	643348518	Borrelia duttonii Ly	1340
46	643692009	Borrelia garinii PBi	1311
47	642555108	Borrelia hermsii DAH	854
48	643348519	Borrelia recurrentis A1	1025
49	642555109	Borrelia turicatae 91E135	853
50	643348520	Buchnera aphidicola 5A (Acyrthosiphon pisum)	590
51	637000043	Buchnera aphidicola APS	615
52	639633012	Buchnera aphidicola Bp	557
53	643348521	Buchnera aphidicola Cc (Cinara cedri)	400
54	637000045	Buchnera aphidicola Sg	588
55	643348522	Buchnera aphidicola Tuc7 (Acyrthosiphon pisum)	588
56	641522608	Burkholderia ambifaria MC40-6	6878
57	637000046	Burkholderia cenocepacia AU 1054	6637
58	639633014	Burkholderia cenocepacia HI2424	7050
59	643348523	Burkholderia cenocepacia J2315	7229
60	641522609	Burkholderia cenocepacia MC0-3	7169
61	637000047	Burkholderia cepacia AMMD	6738
62	643692013	Burkholderia glumae BGR1	5854
63	637000048	Burkholderia mallei ATCC 23344	5500
64	640069303	Burkholderia mallei NCTC 10229	5635
65	640069304	Burkholderia mallei NCTC 10247	5978
66	639633015	Burkholderia mallei SAVP1	5309
67	642555111	Burkholderia multivorans ATCC 17616	6193
68	641228482	Burkholderia multivorans ATCC 17616	6373
69	642555112	Burkholderia phymatum STM815	7574
70	642555113	Burkholderia phytofirmans PsJN	7487
71	640069305	Burkholderia pseudomallei 1106a	7278
72	637000049	Burkholderia pseudomallei 1710b	6436
73	640069306	Burkholderia pseudomallei 668	7322
74	637000050	Burkholderia pseudomallei K96243	5942
75	637000051	Burkholderia sp. 383	7828
76	637000052	Burkholderia thailandensis E264	5727
77	640069307	Burkholderia vietnamiensis G4	7880
78	637000053	Burkholderia xenovorans LB400	9059
79	640753009	Campylobacter concisus 13826	2180
80	640753010	Campylobacter curvus 525.92	2115
81	639633016	Campylobacter fetus fetus 82-40	1823
82	640753011	Campylobacter hominis ATCC BAA-381	1798
83	637000054	Campylobacter jejuni RM1221	1938
84	640753012	Campylobacter jejuni doylei 269.97	2037
85	639633017	Campylobacter jejuni jejuni 81-176	1816
86	640753013	Campylobacter jejuni jejuni 81116	1681

87	637000055	Campylobacter jejuni jejuni NCTC 11168	1705
88	643692014	Campylobacter lari RM2100	1600
89	637000062	Chlamydia muridarum Nigg	957
90	644736341	Chlamydia trachomatis 434/Bu	926
91	637000063	Chlamydia trachomatis A/HAR-13	965
92	644736342	Chlamydia trachomatis B/Jali20/OT	875
93	644736343	Chlamydia trachomatis B/TZ1A828/OT	1002
94	637000064	Chlamydia trachomatis D/UW-3/CX	941
95	644736344	Chlamydia trachomatis L2b/UCH-1/proctitis	925
96	637000065	Chlamydophila abortus S26/3	999
97	637000066	Chlamydophila caviae GPIC	1050
98	637000067	Chlamydophila felis Fe/C-56	1057
99	637000068	Chlamydophila pneumoniae AR39	1156
100	637000069	Chlamydophila pneumoniae CWL029	1096
101	637000070	Chlamydophila pneumoniae J138	1113
102	637000071	Chlamydophila pneumoniae TW-183	1157
103	637000072	Chlorobium chlorochromatii CaD3	2100
104	642555121	Chlorobium limicola DSM 245	2576
105	642555122	Chlorobium phaeobacteroides BS1	2611
106	639633020	Chlorobium phaeobacteroides DSM 266	2805
107	640427130	Chlorobium phaeovibrioides DSM 265	1831
108	637000076	Clostridium acetobutylicum ATCC 824	4022
109	640753016	Clostridium beijerinckii NCIMB 8052	5290
110	640753017	Clostridium botulinum A ATCC 19397	3750
111	640427109	Clostridium botulinum A ATCC 3502	3825
112	640753018	Clostridium botulinum A Hall	3622
113	643692016	Clostridium botulinum A2 Kyoto-F	3978
114	641522617	Clostridium botulinum A3 Loch Maree	4092
115	641522618	Clostridium botulinum B Eklund 17B	3639
116	641522619	Clostridium botulinum B1 Okra	3961
117	643692017	Clostridium botulinum Ba4 657	4206
118	642555125	Clostridium botulinum E3 Alaska E43	3369
119	640753019	Clostridium botulinum F Langeland	3863
120	643348528	Clostridium cellulolyticum H10	3575
121	640069308	Clostridium difficile 630	3983
122	646311914	Clostridium difficile CD196	3565
123	646311915	Clostridium difficile R20291	3599
124	640753020	Clostridium kluyveri DSM 555	4073
125	643348529	Clostridium kluyveri NBRC 12016	3604
126	639633021	Clostridium novyi NT	2485
127	637000079	Clostridium perfringens 13	2905
128	637000077	Clostridium perfringens ATCC 13124	3066
129	642555126	Clostridium perfringens SM101	2748
130	641228486	Clostridium phytofermentans ISDg	3991
131	637000080	Clostridium tetani E88	2565
132	640069309	Clostridium thermocellum ATCC 27405	3335

133	643692018	Corynebacterium aurimucosum ATCC 700975	2617
134	637000082	Corynebacterium diphtheriae NCTC 13129	2395
135	644736345	Corynebacterium efficiens YS-314	3064
136	639279306	Corynebacterium glutamicum ATCC 13032 (Bielefeld)	3211
137	639279307	Corynebacterium glutamicum ATCC 13032 (Kitasato)	3064
138	640427110	Corynebacterium glutamicum R	3174
139	637000085	Corynebacterium jeikeium K411	2186
140	643692019	Corynebacterium kroppenstedtii DSM 44385	2073
141	641522620	Corynebacterium urealyticum DSM 7109	2084
142	643348531	Coxiella burnetii CbuG_Q212	1916
143	643348532	Coxiella burnetii CbuK_Q154	1992
144	640753021	Coxiella burnetii Dugway 7E9-12	2257
145	641228487	Coxiella burnetii RSA 331	2263
146	637000086	Coxiella burnetii RSA 493	2175
147	637000095	Desulfovibrio desulfuricans G20	3874
148	643348538	Desulfovibrio desulfuricans desulfuricans ATCC 27774	2443
149	644736352	Desulfovibrio magneticus RS-1	4760
150	644736353	Desulfovibrio salexigens DSM 2638	3937
151	643348539	Desulfovibrio vulgaris Miyazaki F	3281
152	639633022	Desulfovibrio vulgaris vulgaris DP4	3199
153	637000096	Desulfovibrio vulgaris vulgaris Hildenborough	3642
154	637000097	Ehrlichia canis Jake	985
155	637000098	Ehrlichia chaffeensis Arkansas	1158
156	637000099	Ehrlichia ruminantium Gardel	992
157	639279308	Ehrlichia ruminantium Welgevonden (ARC-OVI)	955
158	639279309	Ehrlichia ruminantium Welgevonden (CIRAD)	976
159	637000104	Escherichia coli 536	4858
160	643348544	Escherichia coli 55989	4985
161	640753023	Escherichia coli APEC O1	5168
162	641522623	Escherichia coli ATCC 8739	4394
163	644736360	Escherichia coli BL21	4087
164	644736361	Escherichia coli BL21(DE3)	4436
165	644736362	Escherichia coli BW2952	4194
166	637000105	Escherichia coli CFT073	5683
167	640753024	Escherichia coli E24377A	5258
168	643348545	Escherichia coli ED1a	5116
169	640753025	Escherichia coli HS	4628
170	643348546	Escherichia coli IAI1	4548
171	643348547	Escherichia coli IAI39	4938
172	641522625	Escherichia coli K12 DH10B	4271

173	646311923	Escherichia coli O103:H2 str. 12009	5312
174	646311924	Escherichia coli O111:H- str. 11128	5610
175	643348548	Escherichia coli O127:H6 E2348/69	4843
176	643348549	Escherichia coli O157:H7 EC4115	5609
177	637000107	Escherichia coli O157:H7 EDL933	5633
178	637000108	Escherichia coli O157:H7 Sakai	5556
179	644736363	Escherichia coli O157:H7 str. TW14359	5500
180	646311925	Escherichia coli O26:H11 str. 11368	5710
181	644736364	Escherichia coli S88	5037
182	643348551	Escherichia coli SE11	5109
183	641522624	Escherichia coli SMS-3-5	5025
184	644736365	Escherichia coli UMN026	5211
185	637000109	Escherichia coli UTI89	5398
186	637000110	Escherichia coli W3110	4427
187	646311926	Escherichia coli str. K-12 substr. MG1655	4497
188	643692022	Escherichia fergusonii UMN026, ATCC 35469	4498
189	637000118	Geobacillus kaustophilus HTA426	3714
190	644736370	Geobacillus sp. WCH70	3597
191	646311929	Geobacillus sp. Y412MC10	6444
192	646311930	Geobacillus sp. Y412MC61	3718
193	640069312	Geobacillus thermodenitrificans NG80-2	3642
194	642555129	Geobacter bemidjiensis Bem	4106
195	642555130	Geobacter lovleyi SZ	3777
196	637000119	Geobacter metallireducens GS-15	3663
197	643348554	Geobacter sp. FRC-32	3894
198	644736371	Geobacter sp. M21	4222
199	637000120	Geobacter sulfurreducens PCA	3552
200	640427115	Geobacter uraniumreducens Rf4	4542
201	637000124	Haemophilus ducreyi 35000HP	1844
202	637000125	Haemophilus influenzae 86-028NP	1913
203	640427116	Haemophilus influenzae PittEE	1757
204	640427117	Haemophilus influenzae PittGG	1798
205	637000126	Haemophilus influenzae Rd KW20	1762
206	643348556	Haemophilus parasuis SH0165	2097
207	637000130	Helicobacter acinonychis Sheeba	1663
208	637000131	Helicobacter hepaticus ATCC 51449	1919
209	637000132	Helicobacter pylori 26695	1624
210	644736374	Helicobacter pylori B38	1427
211	643348558	Helicobacter pylori G27	1547
212	637000133	Helicobacter pylori HPAG1	1590
213	637000134	Helicobacter pylori J99	1541
214	643348559	Helicobacter pylori P12	1620
215	642555131	Helicobacter pylori Shi470	1609
216	637000138	Lactobacillus acidophilus NCFM	1970
217	639633027	Lactobacillus brevis ATCC 367	2324
218	639633028	Lactobacillus casei ATCC 334	2865
219	642555134	Lactobacillus casei casei BL23	3119

220	637000139	Lactobacillus delbrueckii bulgaricus ATCC 11842	2234
221	639633029	Lactobacillus delbrueckii bulgaricus ATCC BAA-365	1865
222	641522633	Lactobacillus fermentum IFO 3956	1915
223	639633030	Lactobacillus gasseri ATCC 33323	1874
224	641228495	Lactobacillus helveticus DPC 4571	1830
225	646311939	Lactobacillus johnsonii FI9785	1803
226	637000140	Lactobacillus johnsonii NCC 533	1941
227	644736381	Lactobacillus plantarum JDM1	3026
228	637000141	Lactobacillus plantarum WCFS1	3230
229	640427118	Lactobacillus reuteri F275	2058
230	642555135	Lactobacillus reuteri F275, JCM1112	1901
231	644736382	Lactobacillus rhamnosus GG	2944
232	644736383	Lactobacillus rhamnosus Lc 705	2992
233	637000142	Lactobacillus sakei sakei 23K	1985
234	637000143	Lactobacillus salivarius salivarius UCC118	2196
235	642555136	Leptospira biflexa Patoc 1 (Ames)	3641
236	642555137	Leptospira biflexa Patoc 1 (Paris)	3770
237	639633032	Leptospira borgpetersenii sv Hardjo-bovis JB197	3193
238	639633033	Leptospira borgpetersenii sv Hardjo-bovis L550	3227
239	637000150	Leptospira interrogans sv Copenhageni Fiocruz L1-130	3707
240	637000152	Listeria innocua Clip11262	3196
241	646311941	Listeria monocytogenes 08-5578	3161
242	646311942	Listeria monocytogenes 08-5923	3039
243	643692027	Listeria monocytogenes 4b CLIP 80459	2851
244	637000154	Listeria monocytogenes 4b F2365	3013
245	637000153	Listeria monocytogenes EGD-e	2991
246	643348561	Listeria monocytogenes HCC23	3059
247	639633035	Listeria welshimeri sv 6b SLCC5334	2913
248	643348563	Methylobacterium chloromethanicum CM4	5847
249	644736386	Methylobacterium extorquens AM1	6294
250	644736387	Methylobacterium extorquens DM4	5829
251	641228497	Methylobacterium extorquens PA1	4939
252	643348564	Methylobacterium nodulans ORS 2060	8885
253	642555139	Methylobacterium populi BJ001	5538
254	641522638	Methylobacterium radiotolerans JCM 2831	6510
255	641522641	Mycobacterium abscessus	4991
256	639633039	Mycobacterium avium 104	5305
257	637000168	Mycobacterium avium paratuberculosis K-10	4415
258	637000169	Mycobacterium bovis AF2122/97	4014

259	639633040	Mycobacterium bovis BCG Pasteur 1173P2	4048
260	643692028	Mycobacterium bovis BCG Tokyo 172	3996
261	640427122	Mycobacterium gilvum PYR-GCK	5683
262	641522642	Mycobacterium marinum M	5501
263	639633041	Mycobacterium smegmatis MC2 155	6941
264	640069320	Mycobacterium sp. JLS	5855
265	639633042	Mycobacterium sp. KMS	6089
266	637000171	Mycobacterium sp. MCS	5704
267	637000172	Mycobacterium tuberculosis CDC1551	4300
268	640427123	Mycobacterium tuberculosis F11	4019
269	640427124	Mycobacterium tuberculosis H37Ra	4099
270	637000173	Mycobacterium tuberculosis H37Rv	4062
271	644736391	Mycobacterium tuberculosis KZN 1435	4107
272	642555140	Mycobacterium ulcerans Agy99	4306
273	639633044	Mycobacterium vanbaalenii PYR-1	6047
274	640427125	Mycoplasma agalactiae PG2	796
275	642555141	Mycoplasma arthritidis 158L3-1	666
276	637000174	Mycoplasma capricolum capricolum ATCC 27343	868
277	644736392	Mycoplasma conjunctivae	725
278	637000175	Mycoplasma gallisepticum R	768
279	637000176	Mycoplasma genitalium G37	525
280	646311946	Mycoplasma hominis	563
281	637000177	Mycoplasma hyopneumoniae 232	728
282	637000178	Mycoplasma hyopneumoniae 7448	714
283	637000179	Mycoplasma hyopneumoniae J	712
284	637000180	Mycoplasma mobile 163K	669
285	637000181	Mycoplasma mycoides mycoides SC PG1	1061
286	637000182	Mycoplasma penetrans HF-2	1075
287	637000183	Mycoplasma pneumoniae M129	733
288	637000184	Mycoplasma pulmonis UAB CTIP	820
289	637000185	Mycoplasma synoviae 53	732
290	637000188	Neisseria gonorrhoeae FA 1090	2081
291	642555143	Neisseria gonorrhoeae NCCP11945	2741
292	641228498	Neisseria meningitidis 053442	2116
293	639633045	Neisseria meningitidis FAM18	2051
294	637000189	Neisseria meningitidis MC58	2226
295	637000190	Neisseria meningitidis Z2491	2227
296	644736394	Neisseria meningitidis alpha14	1943
297	643348568	Pseudomonas aeruginosa LESB58	6026
298	640753042	Pseudomonas aeruginosa PA7	6396
299	637000218	Pseudomonas aeruginosa PAO1	5671
300	639279310	Pseudomonas aeruginosa UCBPP-PA14	5994
301	637000219	Pseudomonas entomophila L48	5293
302	637000220	Pseudomonas fluorescens Pf-5	6257
303	637000221	Pseudomonas fluorescens PfO-1	5857
304	643692031	Pseudomonas fluorescens SBW25	6492
305	640427131	Pseudomonas mendocina ymp	4730

306	640427132	Pseudomonas putida F1	5423
307	641522645	Pseudomonas putida GB-1	5515
308	637000222	Pseudomonas putida KT2440	5481
309	641522646	Pseudomonas putida W619	5292
310	640427133	Pseudomonas stutzeri A1501	4237
311	637000223	Pseudomonas syringae pv. phaseolicola 1448A	5436
312	637000224	Pseudomonas syringae pv. syringae B728a	5245
313	637000225	Pseudomonas syringae pv. tomato DC3000	5721
314	640427137	Rhizobium etli CFN 42	6109
315	642555152	Rhizobium etli CIAT 652	6116
316	644736401	Rhizobium leguminosarum bv. trifolii WSM1325	7292
317	643348569	Rhizobium leguminosarum bv. trifolii WSM2304	6643
318	639633055	Rhizobium leguminosarum bv. viciae 3841	7357
319	643692032	Rhizobium sp. NGR234 (ANU265)	6437
320	639279312	Rhodopseudomonas palustris BisA53	4996
321	637000237	Rhodopseudomonas palustris BisB18	5028
322	637000238	Rhodopseudomonas palustris BisB5	4501
323	637000239	Rhodopseudomonas palustris CGA009	4918
324	637000240	Rhodopseudomonas palustris HaA2	4788
325	642555153	Rhodopseudomonas palustris TIE-1	5377
326	642555154	Salmonella enterica Agona SL483	4720
327	643692035	Salmonella enterica Paratyphi C RKS4594	4743
328	641228505	Salmonella enterica arizonae sv 62:z4,z23:--	4695
329	637000251	Salmonella enterica enterica sv Choleraesuis SC-B67	4996
330	643348572	Salmonella enterica enterica sv Enteritidis P125109	4312
331	643348573	Salmonella enterica enterica sv Gallinarum 287/91	4062
332	637000252	Salmonella enterica enterica sv Paratyphi A ATCC 9150	4295
333	641228506	Salmonella enterica enterica sv Paratyphi B SPB7	5772
334	637000254	Salmonella enterica enterica sv Typhi CT18	5165
335	637000253	Salmonella enterica enterica sv Typhi Ty2	4719
336	642555155	Salmonella enterica sv Dublin CT_02021853	4721
337	642555156	Salmonella enterica sv Heidelberg SL476, CVM30485	4884

338	642555157	Salmonella enterica sv Newport SL254	4913
339	642555158	Salmonella enterica sv Paratyphi A AKU_12601	4208
340	642555159	Salmonella enterica sv Schwarzengrund CVM19633	4730
341	637000255	Salmonella typhimurium LT2	4781
342	639633057	Shewanella amazonensis SB2B	3791
343	640069330	Shewanella baltica OS155	4741
344	640753049	Shewanella baltica OS185	4618
345	641228507	Shewanella baltica OS195	4857
346	643348574	Shewanella baltica OS223	4622
347	637000256	Shewanella denitrificans OS217	3914
348	637000257	Shewanella frigidimarina NCIMB 400	4209
349	641522648	Shewanella halifaxensis HAW-EB4	4462
350	640069331	Shewanella loihica PV-4	4010
351	637000258	Shewanella oneidensis MR-1	4657
352	641228508	Shewanella pealeana ATCC 700345	4434
353	643348575	Shewanella piezotolerans WP3	5047
354	640427141	Shewanella putrefaciens CN-32	4152
355	640753050	Shewanella sediminis HAW-EB3	4666
356	639633058	Shewanella sp. ANA-3	4537
357	637000259	Shewanella sp. MR-4	4098
358	637000260	Shewanella sp. MR-7	4172
359	639633059	Shewanella sp. W3-18-1	4237
360	641522649	Shewanella woodyi ATCC 51908	5085
361	641522650	Shigella boydii CDC 3083-94	4725
362	637000261	Shigella boydii Sb227	4640
363	640427142	Shigella dysenteriae Sd197	4912
364	637000263	Shigella flexneri 2a 2457T	4604
365	637000264	Shigella flexneri 2a 301	4865
366	637000265	Shigella flexneri 5 8401	4466
367	640427143	Shigella sonnei Ss046	4841
368	637000272	Staphylococcus aureus RF122	2690
369	637000273	Staphylococcus aureus aureus COL	2759
370	640753052	Staphylococcus aureus aureus JH1	2935
371	640427145	Staphylococcus aureus aureus JH9	2872
372	643692036	Staphylococcus aureus aureus MRSA USA300_TCH1516	2774
373	637000274	Staphylococcus aureus aureus MRSA252	2844
374	637000275	Staphylococcus aureus aureus MSSA476	2752
375	637000276	Staphylococcus aureus aureus MW2	2747
376	640753053	Staphylococcus aureus aureus Mu3	2776
377	637000277	Staphylococcus aureus aureus Mu50	2843
378	637000278	Staphylococcus aureus aureus N315	2734
379	637000279	Staphylococcus aureus aureus NCTC 8325	3006
380	640753054	Staphylococcus aureus aureus Newman	2726
381	637000280	Staphylococcus aureus aureus USA300	2725

382	646311955	Staphylococcus aureus subsp. aureus ED98	2772
383	643692037	Staphylococcus carnosus carnosus TM300	2538
384	637000281	Staphylococcus epidermidis ATCC 12228	2602
385	637000282	Staphylococcus epidermidis RP62A	2704
386	642555160	Staphylococcus haemolyticus JCSC1435	2809
387	637000284	Staphylococcus saprophyticus saprophyticus ATCC 15305	2637
388	637000286	Streptococcus agalactiae A909	2152
389	637000287	Streptococcus agalactiae NEM316	2257
390	644736406	Streptococcus dysgalactiae subsp. equisimilis GGS_124	2167
391	643692038	Streptococcus equi equi 4047	2095
392	643692039	Streptococcus equi zooepidemicus H70	1961
393	642555163	Streptococcus equi zooepidemicus MGCS10565	1965
394	646311957	Streptococcus gallolyticus UCN34	2312
395	640753055	Streptococcus gordonii Challis CH1	2149
396	637000288	Streptococcus mutans UA159	2059
397	643692040	Streptococcus pneumoniae 70585	2272
398	643348576	Streptococcus pneumoniae ATCC 700669	2079
399	641522651	Streptococcus pneumoniae CGSP14	2276
400	639633061	Streptococcus pneumoniae D39	2090
401	642555164	Streptococcus pneumoniae G54 (MLST ST63)	2185
402	641522652	Streptococcus pneumoniae Hungary19A-6	2222
403	643692041	Streptococcus pneumoniae JJA	2193
404	643692042	Streptococcus pneumoniae P1031	2143
405	637000289	Streptococcus pneumoniae R6	2141
406	637000290	Streptococcus pneumoniae TIGR4	2298
407	643692043	Streptococcus pneumoniae Taiwan19F-14	2114
408	637000291	Streptococcus pyogenes M1 GAS	1830
409	637000292	Streptococcus pyogenes MGAS10270	2091
410	637000293	Streptococcus pyogenes MGAS10394	1992
411	637000294	Streptococcus pyogenes MGAS10750	2085
412	637000295	Streptococcus pyogenes MGAS2096	2000
413	637000296	Streptococcus pyogenes MGAS315	1971
414	637000297	Streptococcus pyogenes MGAS5005	1969
415	637000298	Streptococcus pyogenes MGAS6180	1999
416	637000299	Streptococcus pyogenes MGAS8232	1951
417	637000300	Streptococcus pyogenes MGAS9429	1983
418	640069333	Streptococcus pyogenes Manfredo	1915
419	643348577	Streptococcus pyogenes NZ131	1784
420	637000301	Streptococcus pyogenes SSI-1	1954
421	640069334	Streptococcus sanguinis SK36	2367

422	640427146	Streptococcus suis 05ZYH33	2274
423	640427147	Streptococcus suis 98HAH33	2276
424	644736407	Streptococcus suis BM407	2024
425	644736408	Streptococcus suis P1/7	1905
426	644736409	Streptococcus suis SC84	1979
427	637000302	Streptococcus thermophilus CNRZ1066	2020
428	639633062	Streptococcus thermophilus LMD-9	1820
429	637000303	Streptococcus thermophilus LMG 18311	1994
430	643348578	Streptococcus uberis 0140J	1843
431	641228511	Thermotoga lettingae TMO	2092
432	637000321	Thermotoga maritima MSB8	1915
433	646311964	Thermotoga naphthophila RKU-10	1867
434	643348584	Thermotoga neapolitana DSM 4359	1988
435	640427150	Thermotoga petrophila RKU-1	1865
436	642487181	Thermotoga sp. RQ2	1868
437	643692053	Vibrio cholerae M66-2	3812
438	643692054	Vibrio cholerae MJ-1236	3894
439	637000333	Vibrio cholerae O1 bv eltor N16961	3998
440	640427151	Vibrio cholerae O395	4031
441	640753058	Vibrio harveyi ATCC BAA-1116	6252
442	637000335	Vibrio parahaemolyticus RIMD 2210633	5032
443	646311966	Vibrio sp. Ex25	4676
444	643348587	Vibrio splendidus LGP32	4572
445	637000336	Vibrio vulnificus CMCP6	4665
446	637000337	Vibrio vulnificus YJ016	5202
447	646311967	Xanthomonas albilineans	3172
448	637000342	Xanthomonas axonopodis pv. citri 306	4501
449	642555169	Xanthomonas campestris pv. campestris	4529
450	637000343	Xanthomonas campestris pv. campestris 8004	4346
451	637000344	Xanthomonas campestris pv. campestris ATCC 33913	4254
452	637000345	Xanthomonas campestris pv. vesicatoria 85-10	4800
453	637000346	Xanthomonas oryzae pv. oryzae KACC10331	4300
454	637000347	Xanthomonas oryzae pv. oryzae MAFF 311018	4443
455	642555170	Xanthomonas oryzae pv. oryzae PXO99A	5048
456	640069335	Yersinia enterocolitica enterocolitica 8081	4303
457	641228512	Yersinia pestis Angola	4439
458	637000350	Yersinia pestis Antiqua	4576
459	637000351	Yersinia pestis CO92	4368
460	637000352	Yersinia pestis KIM	4445
461	637000353	Yersinia pestis Nepal516	4306
462	640427152	Yersinia pestis Pestoides F	4359
463	637000354	Yersinia pestis bv Microtus, 91001	4462

464	640753060	Yersinia pseudotuberculosis IP 31758	4529
465	637000355	Yersinia pseudotuberculosis IP 32953	4276
466	642555171	Yersinia pseudotuberculosis PB1/+	4342
467	641522661	Yersinia pseudotuberculosis YPIII	4298
