# Supplementary material for: Genome Majority Vote Improves Gene Predictions
Source: PLoS Comput Biol. 2011 Nov 17;7(11):e1002284. doi: 10.1371/journal.pcbi.1002284 (PMC3219611; doi:10.1371/journal.pcbi.1002284)
Supplement: Table S3 — Source files for GenBank default and Glimmer3 gene start sites for 5 genome sets of medium and high diversity. (PDF) [file pcbi.1002284.s012.pdf]

Supplementary Table 3 for M.E. Wall *et al*., Genome majority vote improves gene predictions, *PLoS Computational Biology* (2011).

	NCBI Directory Name	Genbank Gene Map File	Glimmer3 Gene Map File
Medium Diversity	Citrobacter_koseri_ATCC_BAA-895	NC_000913.ptt.gz	NC_000913.Glimmer3.gz
	Citrobacter_koseri_ATCC_BAA-895	NC_009792.ptt.gz	NC_009792.Glimmer3.gz
	Citrobacter_koseri_ATCC_BAA-895	NC_009793.ptt.gz	NC_009793.Glimmer3.gz
	Citrobacter_koseri_ATCC_BAA-895	NC_009794.ptt.gz	NC_009794.Glimmer3.gz
	ecoli_K12_substr_MG1655	NC_000913.ptt.gz	NC_000913.Glimmer3.gz
	ecoli_K12_substr_MG1655	NC_004547.ptt.gz	NC_004547.Glimmer3.gz
	Klebsiella_pneumoniae_342	NC_011281.ptt.gz	NC_011281.Glimmer3.gz
	Klebsiella_pneumoniae_342	NC_011281.ptt.gz	NC_011281.Glimmer3.gz
	Klebsiella_pneumoniae_342	NC_011282.ptt.gz	NC_011282.Glimmer3.gz
	Klebsiella_pneumoniae_342	NC_011282.ptt.gz	NC_011282.Glimmer3.gz
	Klebsiella_pneumoniae_342	NC_011283.ptt.gz	NC_011283.Glimmer3.gz
	Klebsiella_pneumoniae_342	NC_011283.ptt.gz	NC_011283.Glimmer3.gz
	Salmonella_enterica_arizonae_serovar_62_z4_z23	NC_005126.ptt.gz	NC_005126.Glimmer3.gz
	Salmonella_enterica_arizonae_serovar_62_z4_z23	NC_010067.ptt.gz	NC_010067.Glimmer3.gz
	Shigella_flexneri_2a	NC_004337.ptt.gz	NC_004337.Glimmer3.gz
	Shigella_flexneri_2a	NC_004851.ptt.gz	NC_004851.Glimmer3.gz
	Shigella_flexneri_2a	NC_007712.ptt.gz	NC_007712.Glimmer3.gz
	Shigella_flexneri_2a	NC_007713.ptt.gz	NC_007713.Glimmer3.gz
	Shigella_flexneri_2a	NC_007714.ptt.gz	NC_007714.Glimmer3.gz
	Shigella_flexneri_2a	NC_007715.ptt.gz	NC_007715.Glimmer3.gz
High diversity	ecoli_K12_substr_MG1655	NC_000913.ptt.gz	NC_000913.Glimmer3.gz
	ecoli_K12_substr_MG1655	NC_009792.ptt.gz	NC_009792.Glimmer3.gz
	ecoli_K12_substr_MG1655	NC_009793.ptt.gz	NC_009793.Glimmer3.gz
	ecoli_K12_substr_MG1655	NC_009794.ptt.gz	NC_009794.Glimmer3.gz
	Erwinia_carotovora_atroseptica_SCRI1043	NC_000913.ptt.gz	NC_000913.Glimmer3.gz
	Erwinia_carotovora_atroseptica_SCRI1043	NC_004547.ptt.gz	NC_004547.Glimmer3.gz
	Klebsiella_pneumoniae_342	NC_011281.ptt.gz	NC_011281.Glimmer3.gz
	Klebsiella_pneumoniae_342	NC_011281.ptt.gz	NC_011281.Glimmer3.gz
	Klebsiella_pneumoniae_342	NC_011282.ptt.gz	NC_011282.Glimmer3.gz
	Klebsiella_pneumoniae_342	NC_011282.ptt.gz	NC_011282.Glimmer3.gz
	Klebsiella_pneumoniae_342	NC_011283.ptt.gz	NC_011283.Glimmer3.gz
	Klebsiella_pneumoniae_342	NC_011283.ptt.gz	NC_011283.Glimmer3.gz
	Photorhabdus_luminescens	NC_005126.ptt.gz	NC_005126.Glimmer3.gz
	Photorhabdus_luminescens	NC_010067.ptt.gz	NC_010067.Glimmer3.gz
	Sodalis_glossinidius_morsitans	NC_004337.ptt.gz	NC_004337.Glimmer3.gz
	Sodalis_glossinidius_morsitans	NC_004851.ptt.gz	NC_004851.Glimmer3.gz
	Sodalis_glossinidius_morsitans	NC_007712.ptt.gz	NC_007712.Glimmer3.gz
	Sodalis_glossinidius_morsitans	NC_007713.ptt.gz	NC_007713.Glimmer3.gz
	Sodalis_glossinidius_morsitans	NC_007714.ptt.gz	NC_007714.Glimmer3.gz
	Sodalis_glossinidius_morsitans	NC_007715.ptt.gz	NC_007715.Glimmer3.gz
